# Pumilio directs deadenylation-associated translational repression of the cyclin-dependent kinase 1 activator RGC-32

**DOI:** 10.1093/nar/gky038

**Published:** 2018-01-27

**Authors:** Michèle Brocard, Sarika Khasnis, C David Wood, Claire Shannon-Lowe, Michelle J West

**Affiliations:** 1 School of Life Sciences, University of Sussex, Falmer, Brighton BN1 9QG, UK; 2 Institute of Immunology and Immunotherapy, College of Medical and Dental Sciences, University of Birmingham, Edgbaston, Birmingham B15 2TT, UK

## Abstract

Response gene to complement-32 (RGC-32) activates cyclin-dependent kinase 1, regulates the cell cycle and is deregulated in many human tumours. We previously showed that RGC-32 expression is upregulated by the cancer-associated Epstein-Barr virus (EBV) in latently infected B cells through the relief of translational repression. We now show that EBV infection of naïve primary B cells also induces RGC-32 protein translation. In EBV-immortalised cell lines, we found that RGC-32 depletion resulted in cell death, indicating a key role in B cell survival. Studying RGC-32 translational control in EBV-infected cells, we found that the RGC-32 3′untranslated region (3′UTR) mediates translational repression. Repression was dependent on a single Pumilio binding element (PBE) adjacent to the polyadenylation signal. Mutation of this PBE did not affect mRNA cleavage, but resulted in increased polyA tail length. Consistent with Pumilio-dependent recruitment of deadenylases, we found that depletion of Pumilio in EBV-infected cells increased RGC-32 protein expression and polyA tail length. The extent of Pumilio binding to the endogenous RGC-32 mRNA in EBV-infected cell lines also correlated with RGC-32 protein expression. Our data demonstrate the importance of RGC-32 for the survival of EBV-immortalised B cells and identify Pumilio as a key regulator of RGC-32 translation.

## INTRODUCTION

RGC-32 (*RGCC*, *C13ORF15*) was first described as a gene induced by cell-cycle activation on exposure of rat oligodendrocytes and human aortic endothelial cells to sub-lytic doses of complement C5b9 (1,2). RGC-32 mRNA is expressed in most tissues and is upregulated at the RNA or protein level in tumours of the colon, prostate, bladder, breast, lung and ovaries (3–6). Conversely, RGC-32 is silenced or downregulated in other tumour contexts including multiple myeloma and glioblastoma (7,8). RGC-32 is involved in many cell-type specific processes including angiogenesis, glucose homeostasis, macrophage phagocytosis and T cell growth (9–12). RGC-32 knock-out mice develop normally but are smaller than wild-type littermates due to impaired placental angiogenesis (10). These mice also have reduced adipose tissue mass and as a result, are protected from diet-induced obesity and insulin resistance normally associated with increased RGC-32 expression in these tissues (13). RGC-32 has no homology to other human proteins, but has been shown to be a binding partner and substrate of cyclin-dependent kinase 1 (CDK1), polo-like kinase 1 (PLK1) and Akt and is centrosome-associated during mitosis (1,7,14). *In vitro* studies have demonstrated that RGC-32 binding to CDK1 increases CDK1 activity in a manner dependent on phosphorylation of threonine 91 in a CDK phosphorylation consensus motif in RGC-32 (14). Consistent with a cell-cycle regulatory function, expression of RGC-32 in smooth muscle cells following G1 arrest promotes S- and M-phase entry (14). Knock-down of RGC-32 also prevents complement and growth factor-induced cell-cycle entry and CDK1 activation in aortic endothelial cells (1).

We previously showed that RGC-32 protein is differentially expressed in B cell-lines infected by Epstein-Barr virus (EBV), with its expression depending on the viral gene expression profile of the infected cells (15). EBV is a herpesvirus associated with multiple malignancies including Burkitt's, Hodgkin's and post-transplant lymphoma and nasopharyngeal and gastric carcinoma. The virus immortalises B cells and establishes a latent infection in these cells. Initial B cell growth transformation results in the expression of all EBV latent proteins including six EBV nuclear antigens (EBNAs) and three latent membrane proteins (LMPs). This pattern of latent gene expression is referred to as latency III and is the pattern of latent gene expression observed in EBV-infected lymphoblastoid cell lines (LCLs) generated *in vitro. In vivo*, growth transformed EBV-infected cells displaying the latency III pattern of gene expression successively downregulate their viral gene expression profile to more restricted patterns of gene expression known as latency II and latency I. This allows infected cells to transit through the B cell differentiation pathway and establish viral persistence in memory B cells. This spectrum of latent gene expression patterns is also observed in EBV-associated tumours, presumably reflecting the different origins of the malignant cells. RGC-32 protein expression was only detected in EBV-infected cell lines that displayed the latency III pattern of gene expression, and not in EBV negative cell lines or cell lines displaying the more restricted latency I pattern of gene expression (15), indicating that RGC-32 expression may be associated with initial growth transformation by EBV.

RGC-32 protein expression was also induced when latency III cell lines were created by infecting EBV-negative lymphoma cell-lines with EBV (15). Interestingly, RGC-32 protein expression did not correlate with RGC-32 mRNA expression in these different B cell lines. High-levels of RGC-32 mRNA were present in cells with no detectable RGC-32 protein and in cells expressing RGC-32 protein, mRNA levels were extremely low (15). In both situations, we found that RGC-32 mRNA was associated with multiple ribosomes, indicating that a post-initiation translational repression mechanism was responsible for suppressing RGC-32 protein production in EBV negative and latency I cell lines (15). Given the potent transforming ability of EBV and the well-documented disruption of cell-cycle regulation by EBV latent genes at multiple potential points (16,17), we postulated that upregulation of RGC-32 by EBV could contribute to growth deregulation in infected cells. In support of this theory, and consistent with previous reports of the effects of RGC-32 on cell-cycle regulation, we demonstrated that overexpression of RGC-32 in B cell lines resulted in disruption of the G2/M checkpoint (15).

Translational mechanisms play a key role in regulating the expression of numerous cell-cycle proteins both in the mitotic cell cycle and during meiotic progression in oocytes (18–20). Translational regulation typically involves the binding of specific RNA binding proteins (RBP) or microRNAs (miRNAs) to the untranslated regions of mRNAs. Multiple RNA binding proteins often act in a combinatorial manner and can promote or antagonise regulation by miRNAs. Pumilio proteins are evolutionary conserved members of the PUF (Pumilio and *fem-3* binding factor) RBP family and act together with other RBPs to repress translation and/or promote mRNA degradation (21). PUF family members contain a conserved RNA binding domain comprising eight α-helical repeats, that each recognise one nucleotide of the consensus Pumilio binding element (PBE) UGUANAUA (22–24). Pumilio proteins repress expression of many cell-cycle regulatory proteins, including the CDK1 binding partner cyclin B in multiple organisms (21,25), and a potential functional homologue of RGC-32, the atypical CDK activator, RINGO, in *Xenopus* oocytes (26). Pumilio proteins have been reported to repress translation or regulate message stability through several mechanisms that may not be mutually exclusive. These include deadenylation of poly(A) tails, decapping of the 5′ end of mRNAs and effects on translation elongation (21).

We investigated the role of RGC-32 in the control of B cell proliferation and used EBV-infected cell lines as a model system to study the translational regulation of RGC-32 expression. We show that RGC-32 is required for the growth and survival of EBV-immortalised cell-lines, indicative of a key role in EBV-driven B cell transformation. We demonstrate that the RGC-32 3′UTR is sufficient to direct translational repression of a reporter gene, in a manner dependent on the presence of a PBE located adjacent to the poly(A) signal. Loss of this PBE did not affect the site of mRNA cleavage, but resulted in lengthening of the poly(A) tail. We show that Pumilio 1 binds the RGC-32 3′UTR at lower levels in EBV-infected cells where RGC-32 protein is expressed correlating Pumilio binding with RGC-32 translational repression in cells. We also show that knock-down of Pumilio proteins in cells leads to increased expression of endogenous RGC-32 protein and a corresponding increase in polyA tail length. Our data therefore indicate that the Pumilio-dependent RGC-32 translational repression mechanism involves shortening of poly(A) length. Interestingly, in B cells where RGC-32 translation is repressed, mRNA levels are both high and ribosome-associated indicating that this Pumilio-dependent deadenylation mechanism does not involve mRNA degradation or inhibition of translational initiation.

## MATERIALS AND METHODS

### Plasmid construction

To create the inducible lentiviral RGC-32 shRNA vectors, pairs of primers coding for shRNA 1 (Ind shRNA-R_2 and Ind shRNA–F_2) and shRNA 2 (Ind shRNA-R_4 and Ind shRNA –F_4) (Supplementary Table S1) were annealed and inserted into the BglII and HindIII sites of pENTR-THT III (gift from Dr H. Hochegger). Selected clones were inserted into pGLTR –x-GFP (gift from Dr H. Hochegger) using the Gateway LR Clonase II enzyme kit (Invitrogen).

To generate the short RGC-32 3′UTR construct (psicheck2 RGC32 3′UTR ΔDSE) for luciferase assays, the 3′UTR sequence (based on the NCBI NM_014059.2 cDNA clone) was amplified using the primers MW496 and MW497 (Supplementary Table S1) from cDNA prepared from Mutu I cells. The PCR product was digested and inserted into the XhoI and NotI sites of psicheck2 (Promega). psicheck2 RGC32 3′UTR DSE (containing sequences including the downstream sequence element (DSE)) was generated by amplifying the 3′UTR sequence of RGC-32 in addition to a further 500 bp downstream region from the bacmid RP11–769F14 (Bacpac resources) using the primers MW496 and MW1506 (Supplementary Table S1). The PCR product was digested and inserted into the XhoI and NotI sites of psicheck2. psicheck2 3′UTR-GAPDH was generated by inserting the 3′UTR sequence of GAPDH (synthesized using GeneArt Strings, Invitrogen) into the XhoI and NotI sites of psicheck2. To create psicheck2 RGC-32 ORF (open reading frame) the RGC-32 coding sequence was amplified from cDNA using the primers MW524 and MW525 (Supplementary Table S1). The PCR product was digested and inserted into the XhoI and NotI sites of psicheck2. To create pRTS-1 RGC-32, the RGC-32 open reading frame was amplified by PCR from pFLAG RGC-32 (15) using primers MW592 and MW593 (Supplementary Table S1) to introduce SfiI sites. The PCR product was then cloned into the SfiI sites of pUC19 SfiI (gift from Prof. G. Bornkamm). An SfiI fragment containing the RGC-32 ORF was then excised and cloned into the SfiI sites of pRTS-1 (gift from Prof G. Bornkamm) replacing the luciferase gene (27).

### Site-directed mutagenesis and deletion

The Q5^®^ Site Directed Mutagenesis Kit (New England Biolabs) was used to generate the 3′UTR mutants and deletions according to the manufacturer's instructions. All primers were designed using NEBaseChanger™ software (Supplementary Table S1).

### Cell lines and culture

EBV infection of CD19 positive primary B cells was carried out as described previously (28). Cell lines were maintained as described previously (29) and passaged twice weekly. DG75 is an EBV negative Burkitt's lymphoma cell line (30). Akata (31), Elijah (32) and Mutu I (33) are EBV-positive latency I Burkitt's lymphoma cell lines. Mutu III is a cell line clone derived from Mutu I cells that drifted to a latency III gene expression profile in culture (33). IB4 (31) and GM12878 (obtained from Coriell Cell Repositories) are EBV-immortalised lymphoblastoid cell lines (latency III) generated by infection of resting B cells *in vitro*. To create IB4 stable cell lines, cells were diluted 1:3 one day prior to transfection and 5 × 10^6^ cells were washed and resuspended in 100 μl of Buffer T (Neon Transfection Kit, Invitrogen). 5 μg of pRTS-1 or pRTS-1 RGC-32 was added to the cells to a final volume of 120 μl. Cells were transfected using a 100 μl Neon tip at 1300 V, 30 ms and 1 pulse. Transfected cells were then transferred to a flask containing pre-warmed media (without antibiotics). After 48 h 100 μg/ml hygromycin B (Invitrogen) was added to select for transfected cells. The hygromycin B concentration was increased to 300 μg/ml after 1 week. Stable hygromycin-resistant cell lines were established within 4 weeks of selection that inducibly expressed GFP in the absence (IB4 pRTS-1) or presence of RGC-32 (IB4 pRTS-1 RGC-32).For protein stability experiments, Mutu I and Mutu III cells were diluted to 5 × 10^5^ cells/ml 24 h prior to treatment with 50 or 100 μg/ml cycloheximide (Sigma) or 50 μM MG132 (Sigma). Cells were incubated with inhibitors for up to 24 h and analysed by western blotting.

### Polysome gradient analysis

Where indicated, 250 μg/ml of puromycin was added to 5 × 10^7^ cells for 15 min at 37°C followed by 100 μg/ml of cycloheximide for a further 3 min. Alternatively, 100 μg of Harringtonine or the equivalent volume of DMSO (0.1%) was added for 3 mins before the addition of cycloheximide. Polysomes were purified and analysed as described previously (15).

### Transient transfections and luciferase assays

For B cell 3′UTR reporter assays, 10^7^ DG75 cells were initially transfected with 0.1–2 μg of plasmid using a Gene Pulser II (Biorad) at 230 V, 950 μF. 1 μg of reporter plasmid was used for all subsequent experiments. For miRNA experiments, 2 × 10^4^ HeLa cells were plated in a 96-well plate 24 h prior to transfection with 100 ng of psicheck2 RGC32 3′UTR ΔDSE or psicheck2 RGC-32 ORF in combination with 100 nM of miR-30c, miR-30d or a mutant miR-30c (Invitrogen) using Dharmafect Duo transfection reagent (Dharmacon, GE Healthcare). Cells were harvested after 48 h. For 3′UTR reporter assays in U2OS cells, cells were seeded in 24-well plates at a density of 5 × 10^4^ cells/well 24 h prior to transfection. Cells were transfected with 100 or 200 ng of plasmid using Dharmafect Duo transfection reagent and cells were harvested 48 h after transfection. Luciferase assays were carried out as described previously (29), but the activity of the Renilla luciferase reporter gene was adjusted for transfection efficiency by dividing by the activity of the constitutively expressed firefly luciferase gene, carried on the same psicheck2 plasmid.

### Constitutive RGC-32 silencing

Constitutive silencing of RGC-32 was performed using MISSION lentiviral transduction particles (Sigma) containing shRNA sequences against human RGC-32 (shRNA 3: TRCN0000150738, shRNA 4: TRCN0000151271, shRNA 5: TRCN0000153026, shRNA 6: TRCN0000156253, shRNA 7:TRCN0000157549) in the TRC1.5 lentivector backbone. MISSION pLKO.1-puro-CMV-TurboGFP™ positive control transduction particles were used as a control. 5 × 10^4^ GM12878 or IB4 cells were transfected by spinoculation for 2 h at 1200g at 32°C at either MOI 1 or MOI 5. Transfected cells were selected by addition of 0.5 μg/ml of Puromycin (Sigma) after 3 days and selection was carried out for time periods up to 5 weeks. The number of live cells was determined by counting cells that excluded trypan blue stain (Sigma).

For rescue experiments, IB4 pRTS-1 and IB4 pRTS-1 RGC-32 cell lines were treated with 1 μg/ml doxycycline (Sigma) for 24 hrs to induce the expression of GFP in the absence or presence of RGC-32. 1 × 10^5^ cells were then transduced with a mix of shRNA 5 and shRNA 6 MISSION lentiviral transduction particles or MISSION lentiviral non-mammalian shRNA control transduction particles (SHC002V) at a MOI of 5 as described above. After transduction cells were maintained in 1 μg/ml doxycycline and 300 μg/ml hygromycin B for 4 days followed by the selection of transfected cells for up to 5 days by the addition of 0.5 μg/ml of puromycin (Sigma). Cells were harvested at different time points, washed once in phosphate buffered saline (PBS) and fixed in 0.5% formaldehyde (Sigma). Fixed cells were stored at 4°C until analysed by flow cytometry (BD Accuri) to determine the number of GFP positive cells.

### Inducible RGC-32 silencing and cell sorting

Lentiviral particles were generated using the GLTR system (34) by transfecting HEK-293 FT cells (gift from Dr H. Hochegger) with pLK01 scrambled shRNA (gift from Dr H. Hochegger), pLK01 shRNA RGC32.1, or pLK01 shRNA RGC32.2 and packaging vectors pVSV-G and psPAX 2 (gift from Dr H. Hochegger) using Fugene HD (Promega) in 10 cm dishes. Lentiviruses were harvested by pelleting cells, decanting the culture supernatant and filtering it through a 0.45 μm MillexGP filter (Millipore). The cleared supernatants were aliquoted and 1 ml of lentivirus was added to 5 × 10^6^ GM12878 or IB4 cells. Cells were assayed for the constitutive expression of GFP after 5 days by flow cytometry using a BD FACS Canto or BD Accuri C6 (BD Biosciences). shRNA expression was induced by addition of doxycycline at 0.5 μg/ml to 2 × 10^5^ cells in a 24-well plate. Cells were split after 5 days and then every 2 days. Cells were harvested at different time periods after shRNA induction and the number of GFP positive cells determined by flow cytometry as described above. Sorting of GFP-positive cells was performed using a BDFACSAria (BD Biosciences).

### Pumilio silencing

Pumilio silencing was performed using the ON-TARGET plus siRNA against Pumilio 1 (Dharmacon, GE Healthcare L-014179-00-0005) and Pumilio 2 (Dharmacon, GE Healthcare L-014031-02-0005) using the Neon transfection system (Thermofisher), according to the manufacturer's instructions. Briefly, 5 × 10^6^ cells were transfected with a final concentration of 200 nM or 500 nM of a 1:1 mix of siRNA against Pumilio 1 and 2 or a non-target control using 100 μl tips and electroporated with 1 pulse at 1300 V for 30 ms. GM12878 cells were incubated in antibiotic-free RPMI supplemented with 10% FCS and 1× Glutamax (Gibco) for 2 days before harvesting. Elijah cells were transfected, incubated for 24 h and then re-transfected with siRNAs and harvested after a further 24 h.

### RNA extraction

For cell panel analysis, total RNA was extracted using TriReagent (Sigma) and RNA samples then purified using the RNeasy kit (Qiagen). For extraction of reporter mRNA from luciferase reporter transfections, total RNA was extracted using TriReagent and DNA digested using 10 units of DNase I (Roche) for 15 min at 37°C before quenching by addition of 5mM EDTA. Samples were purified by extraction with acidic phenol–chloroform (Ambion) and RNA then purified using the RNeasy kit.

### RT-QPCR

RNA concentrations were determined using a Nanodrop 2000 (Thermo Scientific) and 1 μg was used to prepare cDNA using the ImProm II reverse transcription kit with random primers (Promega). Quantitative PCR was performed in duplicate using the standard curve absolute quantification method on an Applied Biosystems 7500 real-time PCR machine as described previously (35) using primers for RGC-32 (exon 3 MW86 and MW87 or exon 4–5 MW387 and MW388 (15)), GAPDH (MW84 and MW85) (15)), actin (MW417 and MW418 (15)) β2 microglobulin (MW1447 and MW1448), CD21 (MW1132 and MW1133) and cyclin B (MW1362 and MW1363 (Supplementary Table S1). The efficiency of all primers was determined prior to use and in each experiment and all had amplification efficiencies within the recommended range (90–105%).

### 3′ RACE

Total RNA (0.5 μg) was mixed with 1 μl of 20 μM 3′ RACE primer mix (36) and denaturation carried out at 70°C for 5 min. Samples were then incubated on ice for 5 min before the addition of 15 μl of reverse transcription ImpromII reaction mix (Promega) and incubation at 25°C for 5 min and 42°C for 1 h. cDNA was precipitated by ethanol precipitation in 300 mM NaCl and 1 μg of Glycogen (Roche) overnight at –20°C and pelleted at 13 000 rpm in a Heraeus benchtop microfuge for 30 min at 4°C. Pellets were washed twice in ice-cold 70% ethanol and resuspended in 20 μl of DNase-free water. Nested PCR was performed with RGC-32 specific primers in exon 3 (MW86 (15)) in the first PCR and at the 3′ end of the ORF in exon 4 (MW387 (15)) in the second PCR. This would give a PCR product of the 3′UTR plus 43 nucleotides. For 3′RACE on luciferase mRNAs, three sequential nested PCR reactions were carried out. The first PCR used a primer specific to the 5′end of the Renilla luciferase ORF (MW1422), the second PCR used a primer specific to the 3′ end of the ORF (MW1446) and the third PCR used a primer specific to the 3′ end of the RGC-32 3′UTR (MW1482). PCR products were purified using the Qiaquick PCR purification kit (Qiagen), analysed by agarose gel electrophoresis and sequenced (Eurofins genomics).

### RNA immunoprecipitation experiments

Cells were harvested and passed through a 70 μm filter to obtain a single cell preparation in PBS. 1 × 10^7^ cells were then fixed in 1% formaldehyde for 10 min at room temperature to crosslink protein and RNA. The reaction was quenched with 0.125 M glycine for 5 min, and cells collected by centrifugation at 1500 rpm for 10 min at 4°C. Cells were washed with ice cold PBS, and frozen at –80°C until required. Cell pellets were lysed on ice for 30 min in 250 μl lysis buffer per 10^7^ cells, supplemented with protease inhibitors (Roche) and RnaseOUT (Invitrogen) (50 mM Hepes pH 7.4, 1% NP40, 0.25% NaDOC, 1 mM EDTA, 2 mM DTT, 150 mM NaCl, 5 mM MgCl_2_). Sonication for 5 s at 25% amplitude was then performed on ice. Cell debris was pelleted and discarded and the lysate was diluted to 0.5 mg/ml in IP dilution buffer supplemented with protease inhibitors and RnaseOUT (50 mM Hepes pH 7.4, 1 mM EDTA, 2 mM DTT, 150 mM NaCl, 5 mM MgCl_2_). A 100 μl aliquot of lysate was then retained as an input control at this stage.

Pre-blocked protein G sepharose beads for use in immunoprecipitations were prepared by incubating 500 μl of a 50% protein G Sepharose (Sigma) slurry with incubation buffer supplemented with BSA 0.5% and yeast tRNA 100 μg/ml (Fisher) (50 mM Hepes pH 7.4, 0.2% NP40, 0.05% NaDOC, 1 mM EDTA, 2 mM DTT, 150 mM NaCl, 5 mM MgCl_2_, protease inhibitors) for 30 min at 4°C with rotation. A 45 μl aliquot of 50% protein G Sepharose slurry was diluted in 250 μl of incubation buffer supplemented with BSA 0.5%, and yeast tRNA (100 μg/ml). Following a 10 minute equilibration at room temperature, the beads were incubated overnight with 10 μg of antibody at 4°C with rotation (Goat Anti-Pumilio 1 (Bethyl Labs A 300-201 A) and Goat IgG Isotype Control (Fisher 10087232).

Antibody bound beads were washed three times with 500 μl incubation buffer supplemented with RnaseOUT. Next, the beads were incubated overnight with 0.5 mg of cell extract (1 ml) at 4°C, with rotation. Complexes were then washed three times in IP dilution buffer, with a final resuspension in 500 μl. 100 μl of the complexes were retained for western blot analysis, whilst the remainder were resuspended in 500 μl of reverse buffer (50 mM Tris–HCl pH 7, 5 mM EDTA, 10 mM DTT, 1% SDS, RnaseOUT). At this stage, the 100 μl input control aliquots were also diluted with 100 μl reverse buffer. The immunoprecipitations and inputs were incubated for 1 h at 70°C, to reverse cross links. 5 μl of Proteinase K (10 mg/ml) was added and the sample incubated for 30 min at 50°C. RNA was purified by the addition of 500 μl acidic phenol:chloroform pH 4.5 (Ambion). RNA was further purified over an RNAeasy column (Qiagen), according to the manufacturer's guidelines, and eluted in 30 μl H_2_O. The ImProm-II Reverse Transcription System (Promega) was used to generate cDNA from 4 μl of input and each immunoprecipitated sample, ready for QPCR analysis with RGC-32 (exon 4–5 MW387 and MW388 (15)), cyclin B (MW1362 and MW1363) (Supplementary Table S1) and GAPDH (MW84 and MW85 (15)) specific primers. Percentage input signals for cyclin B and RGC-32 for each Pumilio 1 immunopreciptation were determined from an input standard curve from each cell line. These signals were then divided by the percentage input signal obtained in the IgG control immunoprecipitation to determine the fold enrichment and then normalised to the background fold enrichment for a non target mRNA (GAPDH).

### Extension poly(A) test (ePAT) assay

PolyA tail length assays were carried essentially as described in (37) using the primers shown in Supplementary Table S1. Briefly, 1 μg of total RNA was mixed with 1 μl of 100 μM anchor primer and samples denatured at 80°C for 5 min before being left to cool to room temperature. Klenow labelling reactions were performed in a final volume of 20 μl using the Superscript III kit (Invitrogen), 5 units of Klenow enzyme and 1 μl of RNaseOUT (Invitrogen) at 25°C for 1 h. Klenow was denaturated at 80°C for 10 min and the reactions placed at 55°C for 2 min before addition of 1 μl of Superscript Revertase III (Invitrogen). The reverse transcription reaction was carried for 1 h at 55°C before denaturation of the enzyme at 80°C for 10 min. cDNA was purified by ethanol precipitation overnight at –20°C in 300 mM NaCl with 1 μg of glycogen (Roche). cDNA was pelleted at 13 000 rpm for 30 min at 4°C followed by two washes with ice-cold 70% ethanol. The pellets were air dried and resuspended in 20 μl of DNase free water. Nested PCRs were performed for RGC-32 mRNA using the reverse universal primer MW1538 and primer MW387 in the first round and primer MW1602 in the second round. Nested PCR for GAPDH used the reverse universal primer MW1538 with primer MW1453 in the first round and MW1562 in the second round. For the ePAT assay on luciferase mRNA, nested PCRs were carried out using the reverse universal primer MW1538 and primers in the luciferase ORF (first round; MW1422 and second round; MW1446). A third PCR specific for the RGC-32 3′UTR was then carried out using the universal primer and primer MW1602. A0 control PCR products (the 3′UTR with no polyA tail) were amplified using MW1602 and MW1705 for RGC-32 and MW1562 and MW1563 for GAPDH. PCR products were analysed on a 2% agarose gel.

### Immunoblotting

Exponentially growing cells were pelleted, washed twice in cold PBS and lysed in 1× Gel sample buffer (50 mM Tris–HCl pH 6.8, 4% SDS, 10% glycerol, 1 mM EDTA, 0.01% Bromophenol Blue and 5% β-Mercaptoethanol, minus or plus 300 mM NaCl). SDS-PAGE and immunoblotting were carried out as described previously (29,38). The following antibodies were used for immunoblotting: anti-actin 1:5000 (A-2066, Sigma), anti-RGC-32 as described in (15), anti-Pumilio 1 at 1:5000 (A300-201A, Bethyl Laboratories, Inc.), anti-Pumilio 2 at 1:2000 (A300-202A, Bethyl Laboratories, Inc.), anti-EBNA2 (PE2) at 1:200, anti-EBNA3A (exalpha) at 1:1000, anti-LMP1 (CS1–4) at 1/300, anti-MYC (9E10 culture supernatant) at 1/15. HRP conjugated anti-rabbit and anti-mouse secondary antibodies (Cell Signalling) were used at 1:2000 and HRP conjugated anti-goat secondary antibodies at 1:5000 (DAKO A/S, Denmark). Western blot quantification was carried out using Li-COR Image studio software using images captured using the Li-COR Odyssey Imaging system. Signals were adjusted for background and normalised to the signal for actin.

## RESULTS

### RGC-32 protein expression is induced on EBV infection of primary B cells

**Figure 1.**
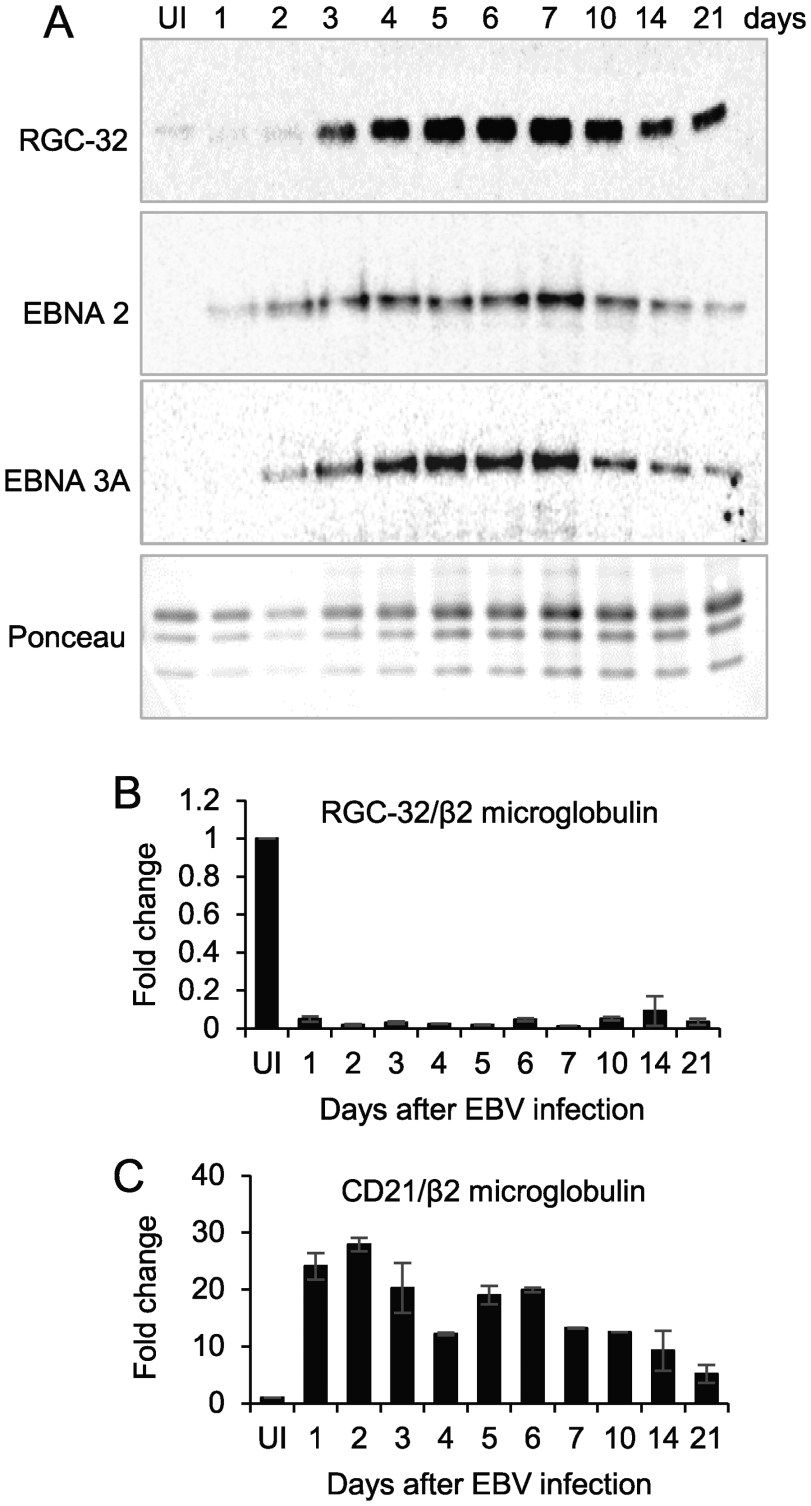
Analysis of RGC-32 expression on EBV infection of naïve B cells. CD19 positive B cells (UI) were infected and samples taken for protein and RNA analysis up to 21 days post-infection. (**A**) Western blot analysis of RGC-32 protein expression and the expression of representative EBV latent proteins (EBNA 2 and EBNA 3A). Ponceau S staining of the membrane was used as a loading control. (**B**) Q-PCR analysis of RGC-32 mRNA levels normalised to levels of β2 microglobulin mRNA and expressed as fold change from uninfected cells. Data show the mean +/- standard deviation of duplicate PCR samples and are representative of two independent experiments. (**C**) Q-PCR analysis of CD21 (CR2) expression as in (B).

We previously showed that RGC-32 protein is expressed in EBV-positive B cell lines displaying the full panel of viral gene expression (latency III), but not in EBV-negative cell lines or EBV-infected cell lines with the more restricted latency I gene expression pattern (15). Since we had also observed that infection of EBV-negative BL cell lines by EBV induced RGC-32 protein expression (15), we examined whether RGC-32 expression was induced in the more physiological setting of EBV infection of naïve resting B cells. Infection of resting B cells leads to the generation of immortalised lymphoblastoid cell lines displaying the latency III pattern of viral gene expression. We found that RGC-32 protein was expressed at very low levels in resting B cells, but was upregulated on EBV infection (Figure 1A), consistent with our previous observations in BL cells (15). Western blotting confirmed that EBV latency III associated proteins were expressed at the expected times post-infection, with EBNA 2 expression detectable early (from day 1) and EBNA 3A expression detectable slightly later (from day 2) (39) (Figure 1A). Interestingly, RGC-32 protein expression began to increase 3 days post-infection coinciding with the point at which EBV-infected cells begin to proliferate rapidly (40).

We previously observed that RGC-32 mRNA expression was very low in EBV-infected latency III cells although RGC-32 protein was expressed (15). This contrasted with the high RGC-32 mRNA levels detectable in cell lines that did not express RGC-32 protein (as a result of a translational block) (15). We found the same inverse relationship between RGC-32 mRNA and protein expression on EBV infection of naïve B cells. RGC-32 mRNA expression decreased rapidly by day 1 post-infection and remained low (Figure 1B). As a control we examined mRNA expression of the EBNA2 activated target gene CD21 (CR2) (41) and found that CD21 mRNA levels were increased as expected from day 1 post-infection (Figure 1C).

These results therefore confirm our previous observations in established cell lines, that EBV infection induces RGC-32 protein expression. They also confirm our observations that RGC-32 mRNA and protein levels do not correlate with one another, with RGC-32 protein expression detected in cells with very low levels of mRNA. In fact, given that RGC-32 mRNA levels decrease by day 1 post-infection and RGC-32 protein expression increases 3 days post-infection, they point to RGC-32 control through distinct transcriptional and post-transcriptional mechanisms.

### RGC-32 expression is required for the survival of EBV immortalised B cells

**Figure 2.**
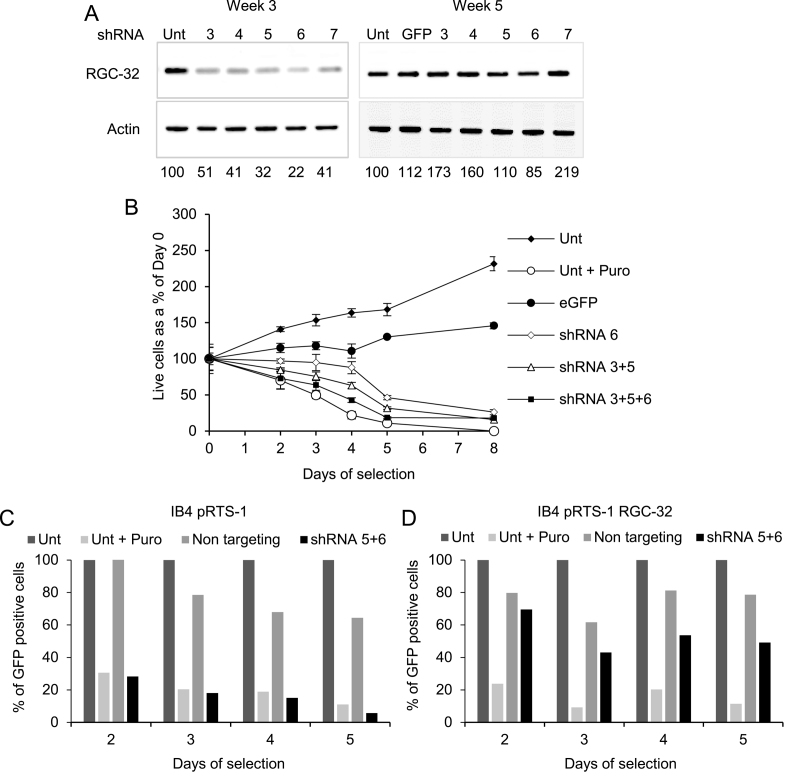
Depletion of RGC-32 mRNA using lentiviruses constitutively expressing shRNAs. (**A**) Western blot analysis of RGC-32 protein expression in GM12878 cells in untransduced cells (Unt) or in transduced cells after 3 or 5 weeks of selection in puromycin. Actin was used as a loading control. Cells were transduced at a MOI of 1 with Mission (Sigma) lentiviral particles carrying shRNAs targeting RGC-32 (shRNA 3 to 7) or the GFP coding sequence (eGFP) as a control. Numbers under the panels show quantification of RGC-32 western blot signals normalised to actin and expressed relative to the untransduced control. (**B**) Numbers of live cells determined by trypan blue exclusion and cell counting at the indicated times following transduction of IB4 cells with Mission lentiviral particles at a MOI of 5 and selection of the transduced cells using puromycin. Data show the mean of four independent cell counts ± standard deviation and are expressed relative to the numbers of live cells at day 0. (**C**) Analysis of the proportion of GFP positive cells present in cultures of IB4 cells stably expressing a control doxycycline inducible GFP expressing plasmid (pRTS-1) following lentiviral transduction and during puromycin selection. Cells were cultured in doxycycline for 24 h prior to transduction with either non-targeting shRNA expressing lentiviruses or a mix of RGC-32 targeting lentiviruses (shRNA 5+6) at an MOI of 5. Untransduced cells and untransduced cells cultured in the presence of puromycin were used as controls. The number of GFP positive cells in lentivirus transduced cells was expressed relative to the number of GFP positive cells in untransduced samples at each time point. (**D**) The proportion of GFP positive cells present in cultures of IB4 cells stably expressing a doxycyclin inducible GFP and RGC-32 expressing plasmid (pRTS-1 RGC-32) following lentiviral transduction and during puromycin selection.

To determine whether the expression of RGC-32 was required for the growth of EBV-immortalised LCLs, we sought to silence RGC-32 expression in EBV-infected LCLs using an RNA interference approach. Since B cell lines are difficult to transfect with plasmids at high efficiency, we used lentiviruses to express RGC-32 targeting shRNAs. Initially, we transduced an EBV-immortalised cell line with puromycin-selectable lentiviruses (Sigma) designed to constitutively express 5 different shRNAs (shRNAs 3 to 7). At a multiplicity of infection (MOI) of 1, after 3 weeks of selection we observed decreases in the expression level of RGC-32 protein by up to 78% with different shRNAs (Figure 2A). However, this reduction in RGC-32 protein expression was not sustained in selected cells. When cells were selected for 5 weeks, those transduced with shRNA-expressing lentiviruses displayed levels of RGC-32 protein expression higher than those in untransduced cells for all shRNAs other than shRNA 6 (85% of control) (Figure 2A). In these experiments we used a control lentivirus expressing GFP, since the non-targeting control shRNA-expressing lentivirus initially supplied by the manufacturer displayed high levels of non-specific toxicity in all cell lines tested and no alternative was available at that time. The loss of RGC-32 knock-down in these longer-term selected cell populations could indicate the loss of cells with reduced RGC-32 expression from the cultures due to cell death. Consistent with this possibility, our initial analysis after four days of drug selection found that the number of shRNA transduced cells remaining was 52%, 67% and 77% of the eGFP control cells for shRNAs 5, 6 and 3 respectively. To explore this further, we used combinations of RGC-32 targeting shRNA expressing lentiviruses and increased the MOI to achieve higher transduction efficiencies.

We observed that following the transduction of cells at a MOI of 5 with RGC-32 targeting lentiviruses, the number of live cells in the culture decreased rapidly during selection for up to 8 days, with increased cell death observed when increasing numbers of RGC-32 targeting lentiviruses were combined (Figure 2B). These data therefore indicate that silencing of RGC-32 leads to the death of EBV-immortalised cells. To confirm that these effects were due to the depletion of RGC-32 and not the result of non-specific toxicity, we performed a rescue experiment by generating an EBV-immortalised cell line that could be induced to overexpress RGC-32 mRNA and thus squelch the effects of RGC-32 targeting shRNAs. We were not able to express a ‘resistant’ form of RGC-32 mRNA as the two most efficient RGC-32 shRNAs (shRNA 5 and 6) target different regions of the RGC-32 open reading frame. Cells were stably transfected with a plasmid containing a doxycycline-inducible bidirectional promoter that drives GFP expression in one direction and RGC-32 expression in the other direction (pRTS-1 RGC-32) (Supplementary Figure S1) (27). A control cell line was also generated using the control pRTS-1 plasmid that drives inducible GFP expression in one direction and firefly luciferase expression in the other direction (pRTS-1) (Supplementary Figure S1). We treated both cell lines with doxycycline for 24 h to induce GFP expression in the absence or presence of RGC-32 expression and then transduced cells with lentiviruses expressing non-targeting (newly supplied by the manufacturer) or RGC-32 targeting shRNAs. We then determined the numbers of surviving GFP positive cells by flow cytometry during positive selection of transduced cells using puromycin and compared this to untransduced cells (Figure 2C and D). Consistent with our previous results (Figure 1B), we found that in the pRTS-1 control cell line expressing endogenous RGC-32 there was a large reduction in the numbers of GFP positive cells present in cultures transduced with RGC-32 targeting shRNAs during selection (Figure 2C). In contrast, GFP positive cell numbers were maintained at high levels in cells transduced with non-targeting lentiviruses (Figure 2C). However, cells co-expressing GFP and exogenous RGC-32 (pRTS-1 RGC-32) transduced with RGC-32 targeting lentiviruses survived much better during selection (Figure 2D). In cells where exogenous RGC-32 expression was induced the proportion of GFP positive cells was 50% of those in untransduced cells after 5 days of selection with RGC-32 targeting shRNAs, compared to 6% in the control cell line (Figure 2C and D). Exogenous expression of RGC-32 therefore protects cells from cell death induced by RGC-32 targeting shRNAs indicating that the effects of these shRNAs are specific.

**Figure 3.**
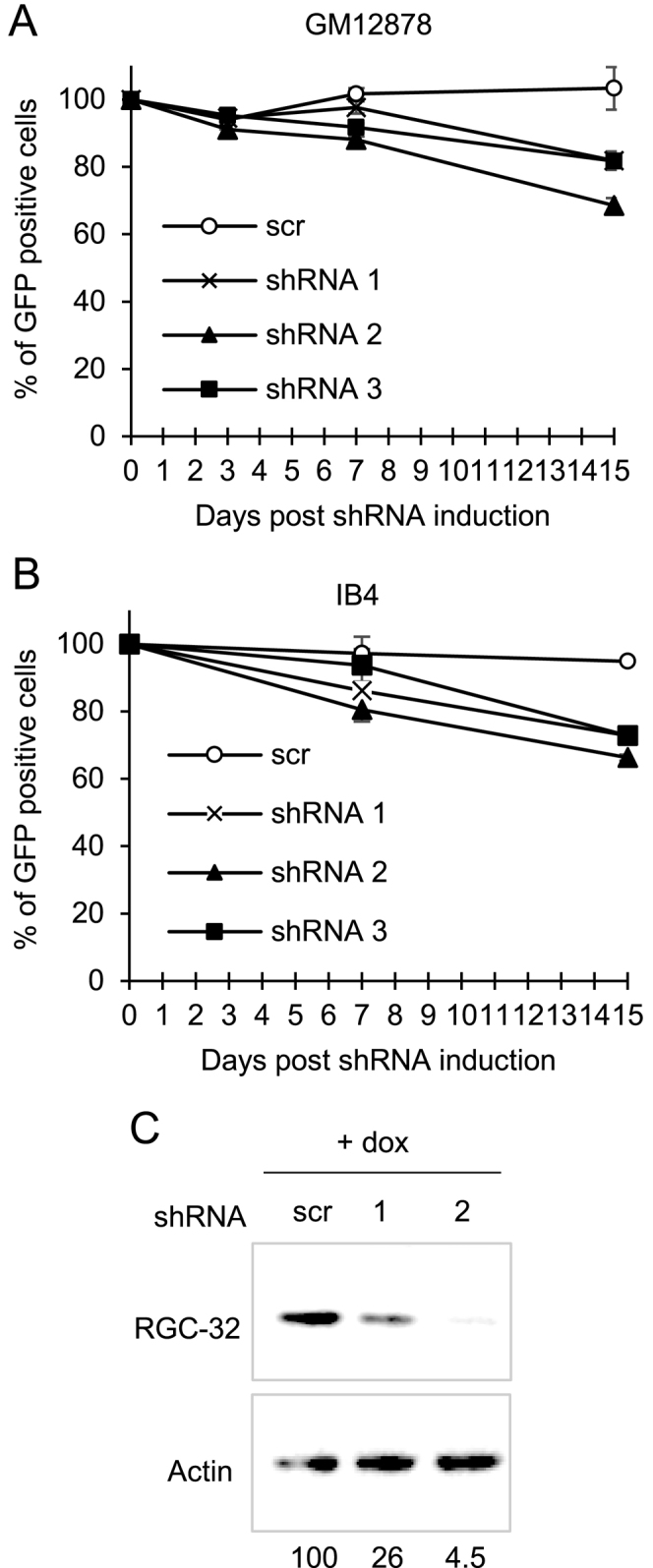
Depletion of RGC-32 mRNA using lentiviruses inducibly expressing shRNAs. (**A**) Flow cytometry analysis of the numbers of GFP-positive GM12878 cells in a time course following doxycycline-induced expression of RGC-32 targeting shRNAs (shRNA 1–3) or a control scrambled shRNA in cells transduced with lentiviruses that also constitutively express GFP. Data show the mean of two biological replicates ± standard deviation and are expressed relative to the number of GFP positive cells following transduction but prior to doxycycline shRNA induction (day 0). (**B**) Analysis of the numbers of GFP-positive IB4 cells following induction of shRNA expression as in (A). (**C**) Western blot analysis of RGC-32 and actin protein levels 4 days after doxycycline induction. GM12878 cells were transduced with scrambled or RGC-32 targeting shRNAs (shRNA 1 or 2) and sorted to enrich the GFP positive population by flow cytometry. Numbers show quantification of RGC-32 western blot signals from GFP positive sorted cells normalised to actin and expressed relative to the scrambled control.

To provide further confirmation of these results, we also created lentiviruses that express doxycycline-inducible shRNAs and constitutively express GFP. We could then monitor cell survival again by determining GFP positive cell numbers at times after shRNA induction. Again, flow cytometry detected a decline in the numbers of GFP-positive cells in the cultures of two EBV-immortalised LCLs (GM12878 and IB4) following doxycycline treatment of cells transduced with lentiviruses expressing shRNAs against RGC-32 (1, 2 and 3) (Figure 3A and B). In contrast, induced expression of a control scrambled shRNA had no effect on the percentage of GFP-positive cells (Figure 3A and B). After enrichment of transduced GM12878 cultures for GFP-positive cells by cell sorting, we confirmed that RGC-32 protein expression was successfully downregulated in cells expressing RGC-32-targeting shRNAs. RGC-32 protein expression was reduced to 26% and 4.5% of control levels following the induction of RGC-32 shRNA 1 and 2, respectively (Figure 3C). Taken together, the results of our depletion experiments demonstrate that RGC-32 expression is required for the survival of EBV-immortalised LCLs.

### RGC-32 mRNA is associated with active ribosomes

We previously demonstrated that despite a lack of RGC-32 protein expression in EBV-infected latency I cells, RGC-32 mRNA was still found within the polyribosome fraction (polysomes) of cytoplasmic mRNA complexes isolated by sucrose gradient centrifugation (15). Since we confirmed that RGC-32 regulation on primary EBV infection mirrors the regulation of RGC-32 detected in latency I and latency III EBV-infected cell lines, we continued our studies into the post-transcriptional regulation of RGC-32 expression, using these cell lines as a model system. Our initial experiments addressed whether the polysomal fractions with which RGC-32 mRNA was associated in latency I cells lacking RGC-32 protein expression, represented active polysomes. We therefore treated latency I and latency III cells with the translation elongation inhibitor, puromycin, which causes premature peptide chain termination and ribosome dissociation. We found that RGC-32 mRNA and a control mRNA (GAPDH) were no longer associated with large polysomes in either cell type following puromycin treatment (Supplementary Figure S2). This is consistent with the presence of active ribosomes associated with RGC-32 mRNA in both latency I and latency III cells. We confirmed these results using the translation inhibitor, harringtonine. Harringtonine blocks the transition from initiation to elongation but does not inhibit initiation or ongoing elongation (polysome run-off). During brief harringtonine treatment, elongating polysomes will therefore complete translation and newly initiating ribosomes will stall near the translation start site. We found that harringtonine treatment led to the loss of polyribosome associated RGC-32 mRNA in both latency I and latency III cells (Supplementary Figure S3). These data indicate that even in latency I cells, RGC-32 mRNA associates with ribosomes that are capable of completing elongation.

We had previously been unable to detect any RGC-32 protein expression in cell lysates from EBV negative and latency I cell lines using standard whole cell lysate preparation techniques. The detection of actively elongating RGC-32 mRNA polysomes in latency I cells however, prompted us to re-address whether we could detect even low levels of RGC-32 protein in these cell lines using alternative protein solubilisation techniques. We found that in the presence of high salt, RGC-32 protein was detectable at low levels in latency I cells and at even higher levels in latency III cells (Supplementary Figure S4). These data therefore indicate that some RGC-32 protein is produced in latency I cells, but that levels are very low and inconsistent with the high level of mRNA.

We next explored the possibility that RGC-32 protein is produced in latency I cells but is either unstable or actively degraded. We had previously treated latency I cells with the proteasome inhibitor MG132 and found that this did not lead to the appearance of detectable RGC-32 protein expression. This indicates that proteasome-mediated degradation is not responsible for the discrepancy between mRNA and protein expression (15). We repeated this experiment using our improved solubilisation technique and again found that MG132 treatment did lead to an increase in RGC-32 protein expression (Supplementary Figure S5C). We also examined the half-life of the RGC-32 protein in both latency III and latency I cell lines by blocking protein synthesis using cycloheximide and monitoring RGC-32 protein levels over time. We found that RGC-32 was stable up to 24 h after cycloheximide treatment in Mutu III latency III cells (Figure S5A). In parallel, we determined the stability of two short half-life proteins, myc and the EBV latent membrane protein 1 (LMP1). Levels of both proteins reduced as expected over the course of the experiments with half-lives of 2.5 and 8 h respectively, similar to published reports (42,43). Latency I cells are more susceptible to apoptosis than latency III cells, since the expression of latency III associated EBV proteins provides protection from apoptosis. It was therefore only possible to treat latency I cells for up to 8 h with the concentrations of cycloheximide required to block protein synthesis. However, we observed no loss of RGC-32 protein over this period (Supplementary Figure S5B). A reduction in RGC-32 protein stability or proteasomal degradation does not therefore appear to be responsible for the low levels of RGC-32 protein in EBV infected latency I cells.

### The RGC-32 3′ untranslated region represses translation

**Figure 4.**
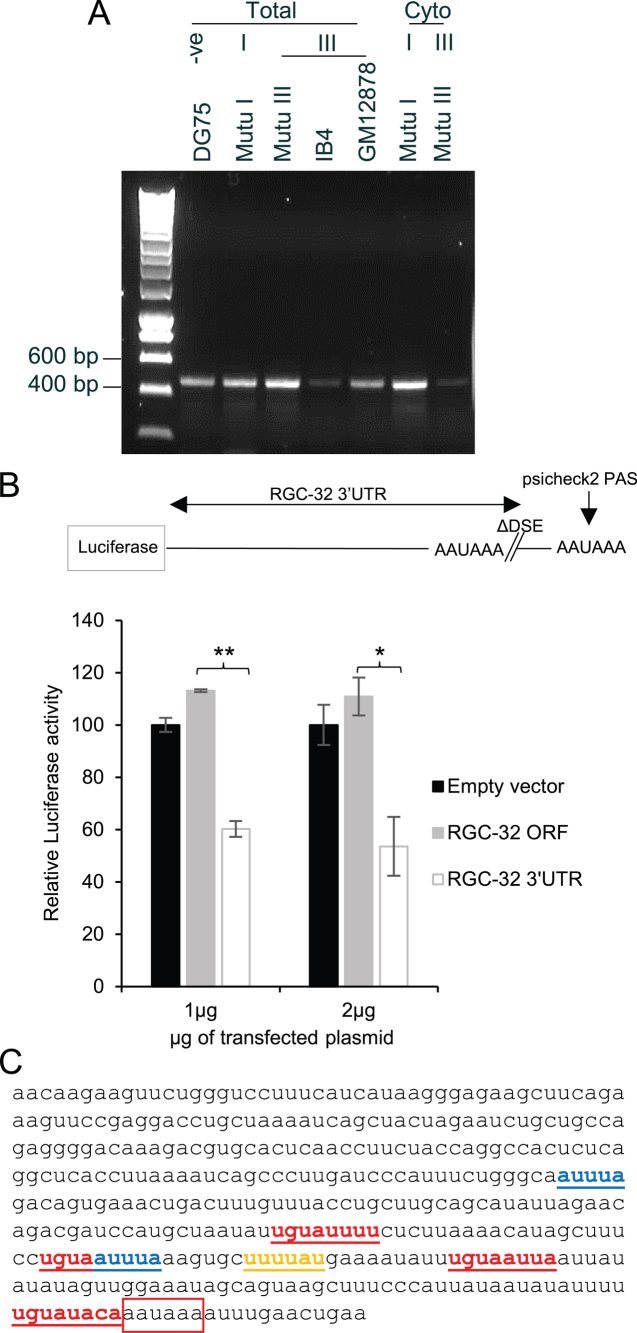
Analysis of the RGC-32 3′UTR. (**A**) 3′RACE analysis of RGC-32 total or cytoplasmic mRNA from a panel of EBV negative and EBV-positive latency I (I) or latency III (III) B cell lines. (**B**) Luciferase reporter assays carried out using constructs containing the RGC-32 3′UTR cloned downstream of the Renilla luciferase gene in the psicheck2 vector. The 3′ RGC-32 gene sequence cloned into the reporter lacks the U-rich and GU-rich downstream sequence element (DSE) present in the endogenous RGC-32 gene, so the synthetic PAS in the psicheck2 vector drives polyadenylation and cleavage. 1 or 2 μg of the construct was transfected into the EBV-negative DG75 BL cell line and luciferase assays carried out after 48 h. Data show Renilla luciferase signals normalised to the internal Firefly luciferase control and are expressed relative to the psicheck2 empty vector Renilla luciferase signal. The mean of two biological replicates ± standard deviation is shown. Student's t-test for the 3′UTR containing reporter construct compared to the empty vector control show P < 0.001 (**) and P < 0.05 (*). (**C**) RNA sequence of the 386 nucleotide 3′UTR of RGC-32 mRNA derived from sequencing of the 3′RACE assay products. Putative AU-rich elements (blue), Pumilio binding elements (red) and a cytoplasmic polyadenylation element (yellow) are shown. The endogenous PAS is boxed in red.

Since our data thus far were consistent with translational control of RGC-32 expression in EBV infected cells, we investigated the potential roles of RGC-32 mRNA untranslated regions (UTR) in regulating RGC-32 expression. RGC-32 mRNA has 5′ and 3′UTRs of approximately 300 and 400 nts, respectively. However, since polysome gradient analysis indicated that there was not a block to RGC-32 translation initiation in latency I cells and 5′UTR directed effects on translation normally affect initiation, we focused our analysis on the RGC-32 3′UTR. *Cis*-acting sequences in mRNA 3′UTRs have been previously shown to have the ability to direct translational repression at post-initiation steps, where it appears that RGC-32 translation is controlled in latency I cells. We first examined whether there were any differences in 3′UTR length between EBV negative, latency I or latency III cells that could be responsible for differential RGC-32 translation. 3′RACE experiments detected a single RGC-32 3′UTR product of identical size in both total and cytoplasmic RNA samples in all cell lines tested corresponding to a 3′UTR length of 386 nts (Figure 4A and C). This is 9 nts shorter than the 3′UTR recorded for the isoform of RGC-32 (AF036549) (14) that we previously demonstrated was the only RGC-32 isoform expressed in B cell lines (15). Sequence analysis of the 3′RACE products found no nucleotide sequence differences and confirmed the use of the same polyadenylation signal and the presence of the same cleavage site across cell lines (data not shown).

We next investigated whether the 3′UTR of RGC-32 could direct translational repression. We cloned the RGC-32 3′UTR downstream of a Renilla luciferase reporter gene and transiently transfected this construct into the EBV-negative cell line, DG75. As a control, we also cloned the similar length sequence of the RGC-32 open reading frame (ORF) downstream of the luciferase gene. In these constructs, polyadenylation and cleavage were directed by the synthetic polyadenylation signal present in the reporter construct, since we did not include the downstream sequence element (DSE) required for usage of the endogenous polyadenylation signal in the RGC-32 3′UTR (Figure 4B). Our results demonstrated that the presence of the RGC-32 3′UTR resulted in a 40–46% decrease in luciferase expression in B cells compared to the empty vector and approximately 50% inhibition relative to the ORF control (Figure 4B). We consistently observed a 40–50% inhibition of luciferase expression in B cells using varying amounts of plasmid DNA (0.1, 0.5, 1 and 2 μg) (Figure 4B and data not shown) indicating that repression was reproducible. We also carried out luciferase reporter assays in the osteosarcoma cell line U20S and detected a 54% and 56% inhibition of luciferase expression compared to the ORF using 0.1 and 0.2 μg of plasmid, respectively. Our data therefore point to the 3′UTR of RGC-32 as a key controller of RGC-32 translation and indicate that repression of RGC-32 expression by the 3′UTR may not be B cell specific.

### A Pumilio binding element in the RGC-32 3′UTR is required for gene repression

To identify putative *cis* acting motifs that could be responsible for translational repression mediated by the RGC-32 3′UTR, we carried out *in silico* analysis. We identified two AU-rich elements, four putative Pumilio binding elements (PBE), and one cytoplasmic polyadenylation element (Figure 4C). AU-rich elements can be bound by multiple factors to regulate message stability and degradation (44), so we examined the potential role of these elements in the repression of gene expression by the RGC-32 3′UTR using a mutagenesis approach. We found that mutation of the two AU-rich elements, either individually or together, had no effect on RGC-32 3′UTR-mediated repression of reporter gene expression (Supplementary Figure S6).

**Figure 5.**
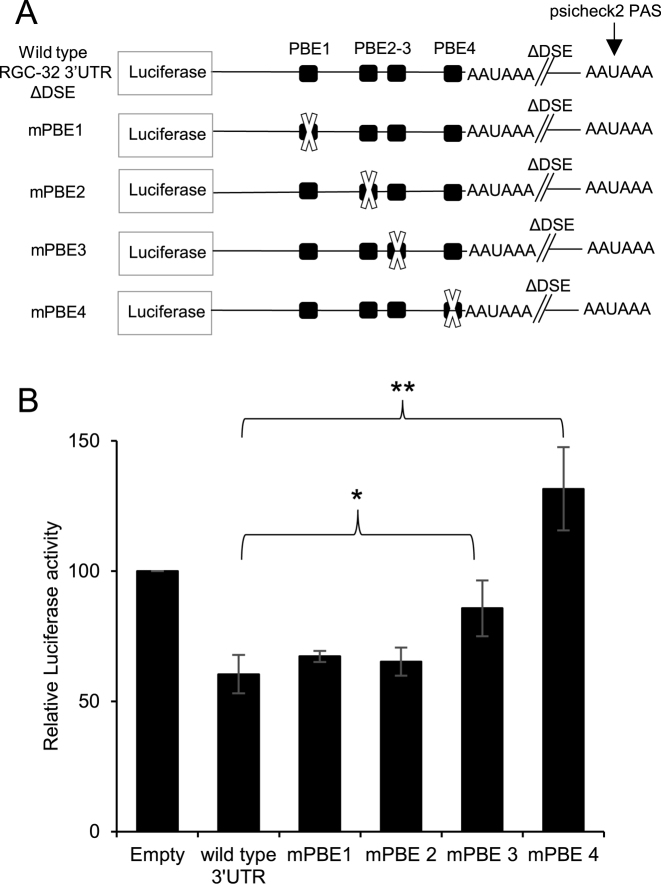
The role of Pumilio binding elements in 3′UTR-mediated repression. (**A**) Diagram showing the Renilla luciferase reporter constructs generated by site-directed mutagenesis of the wild-type RGC-32 3′UTR construct. Each of the four putative PBEs was mutated to generate mPBE1–4. (**B**) Luciferase reporter assays carried out by transfecting 1 μg of each construct into DG75 cells. Results show the mean ± standard deviation of four independent experiments normalised as in Figure 4. Student's *t*-test for mutants compared to the wild-type 3′UTR show *P* < 0.001 (**) and *P* < 0.05 (*).

Since Puf family proteins are known translational repressors of cell-cycle and survival mRNAs (21), we next examined the role of the PBEs in 3′UTR-mediated repression. All of the putative PBEs contain the conserved 5′ core of the 8 nucleotide Pumilio binding motif (45) (UGUA) with some sequence variation 3′ to the core, as previously described (23) (Figure 4C). PBE1 and PBE2 have mismatches at positions 6 and 8 of the PUM consensus motif (UGUANAUA), PBE3 has a mismatch at position 6 and PBE4 has a mismatch at position 7. Analysis using the Targetscan database (http://www.targetscan.org/vert_71/) indicates that the sequences of PBE1, PBE2 and PBE3 are completely conserved across species and PBE4 is well conserved (some species have a G in place of A at position 6). We mutated the conserved core sequence UGUA of each of the four PBEs individually and examined the effect on RGC-32 3′UTR-mediated repression (Figure 5A). Although mutation of PBEs 1 and 2 had no effect on the ability of the RGC-32 3′UTR to repress translation, mutation of PBE4 completely abolished repression (Figure 5B). Mutation of PBE3 reduced repression to approximately half that observed with the wild-type 3′UTR (Figure 5B). These data therefore indicate that PBE4 is required for the repression of gene expression by the RGC-32 3′UTR and that PBE3 may also play a role. The fact that PBE4 and PBE3 affect 3′UTR repression activity is consistent with the better matches of these putative elements to the Pumilio consensus motif.

**Figure 6.**
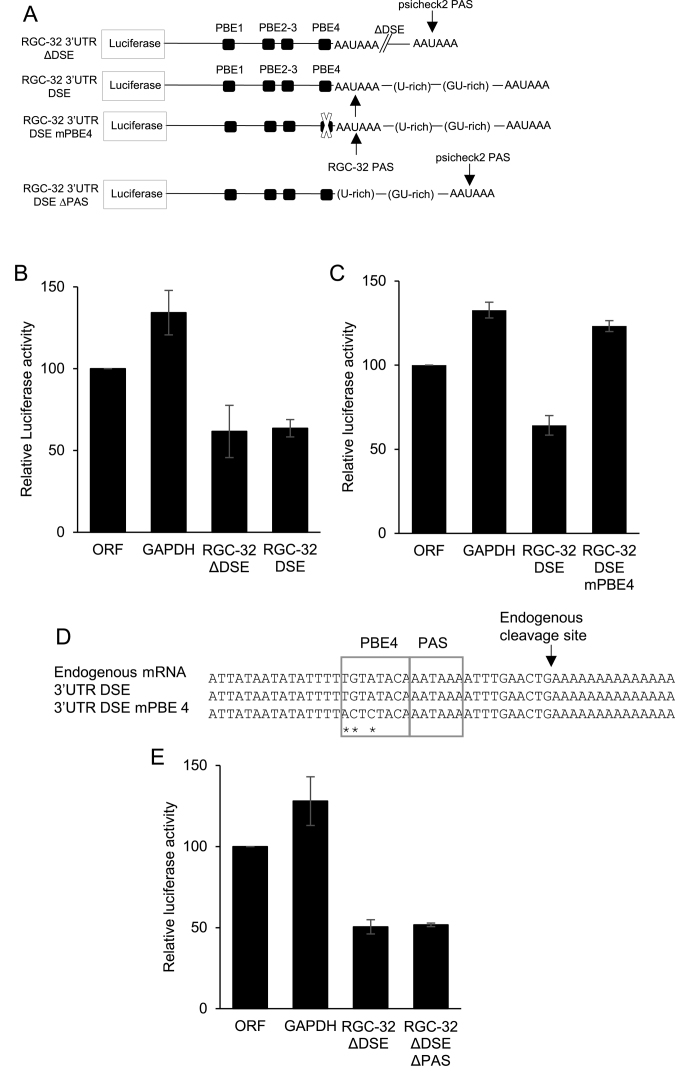
Analysis of the role of PAS location in PBE-dependent repression via the 3′UTR. (**A**) Diagram showing the Renilla reporter construct generated containing the additional U-rich and GU-rich downstream sequence element (DSE) located 3′ of the endogenous RGC-32 PAS and required for its usage. (**B**) Luciferase reporter assays carried out as in Figure 5 to compare repression by the RGC-32 3′UTR when either the endogenous or downstream psicheck2 PAS are used. Control plasmids containing the RGC-32 ORF sequence or the GAPDH 3′UTR cloned downstream of the Renilla luciferase gene were used. Data show Renilla luciferase signals normalised to the internal Firefly luciferase control and are expressed relative to the psicheck2 ORF luciferase signal. The mean of two biological replicates -/+ standard deviation is shown. (**C**) Luciferase reporter assays analysing the effect of mutating PBE4 when the endogenous RGC-32 PAS is used. (**D**) Sequence alignment of the products of a 3′RACE assay performed on the Renilla luciferase mRNA from the transfection shown in (C), compared to the sequence obtained from 3′RACE analysis of the endogenous RGC-32 mRNA. (**E**) Luciferase reporter assays using constructs where the endogenous RGC-32 PAS has been deleted in the context of the wild-type 3′UTR the mPBE4 3′UTR.

Interestingly, the PBE4 motif in the RGC-32 3′UTR is located adjacent to the endogenous polyadenylation signal (PAS) (Figure 4C). In fact PBE4 resembles a polyadenylation upstream element (USE) known to interact with the cleavage factor Im (CFIm) and to be required for the proper recognition of the PAS by the cleavage and polyadenylation factor (CPSF) (46,47). In the 3′UTR reporter experiments described above, the endogenous PAS of RGC-32 was not used, with polyadenylation and cleavage directed by the synthetic PAS of the plasmid. We therefore investigated whether PBE4 was able to direct 3′UTR-mediated repression when the endogenous PAS of RGC-32 was active and cleavage and polyadenylation factors were bound. An additional 3′ region of the RGC-32 gene that included a U-rich and GU-rich DSE was therefore inserted into the reporter construct to stimulate the use of the endogenous PAS (Figure 6A). Using 3′RACE and sequencing, we confirmed that the insertion of the DSE led to usage of the endogenous PAS and cleavage of the message at the same site as the endogenous RGC-32 mRNA isolated from cells (Figure 6D). We found that the insertion of the additional downstream sequence and the use of the endogenous PAS did not affect the ability of the RGC-32 3′UTR to repress reporter gene expression (Figure 6B).

We next mutated PBE4 in the context of the longer RGC-32 3′UTR reporter construct. Again, we found that mutation of PBE4 led to a loss of RGC-32 3′UTR-mediated repression (Figure 6C). Importantly, mutation of PBE4 did not impair the usage of the endogenous PAS, since the RGC-32 3′UTR was cleaved at the same site as the wild-type 3′UTR containing construct and the endogenous message (Figure 6D). PBE4 sequences do not therefore appear to be required for the recognition or function of the endogenous RGC-32 PAS. In fact, we found that RGC-32 3′UTR-mediated repression was maintained when the endogenous PAS was deleted, but PBE4 was left intact (Figure 6E).

Taken together, these results indicate that PBE4 is the main mediator of repression directed by the RGC-32 3′UTR in this reporter assay. Importantly, despite its proximity to the endogenous PAS, this PBE appears to function independently of the PAS and can direct repression even when a downstream PAS is used.

**Figure 7.**
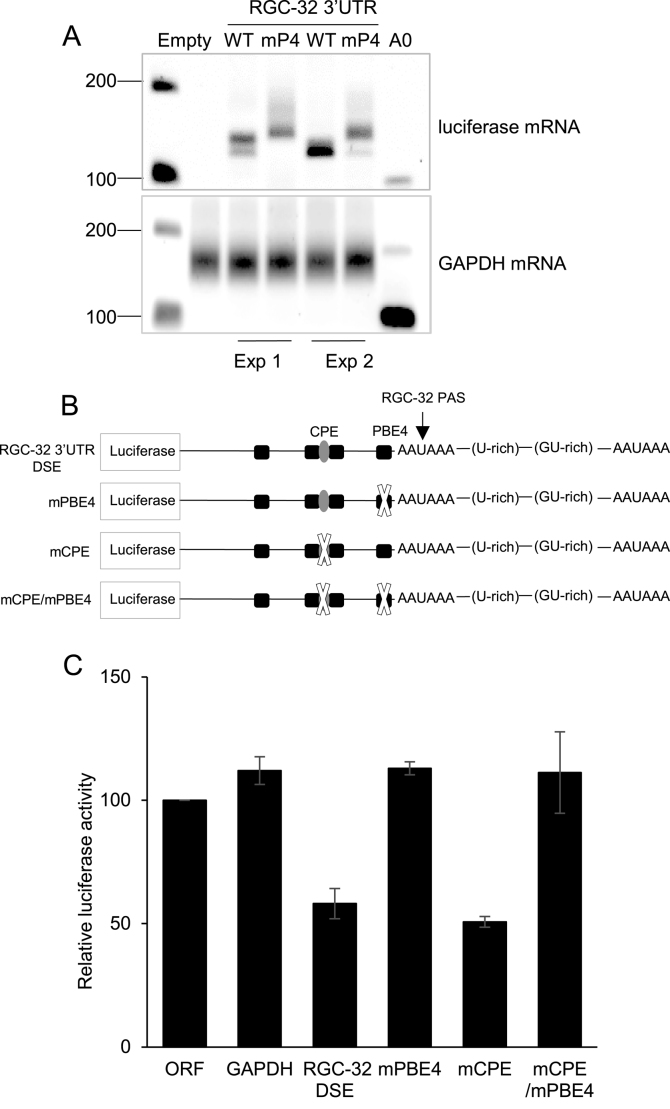
Analysis of the effects of RGC-32 3′UTR-mediated repression on polyadenylation. (**A**) ePAT assay to measure poly A tail length performed on Renilla luciferase mRNAs carrying the wild-type RGC-32 3′UTR or the PBE4 mutant 3′UTR from the experiments shown in Figure 6C. Control ePAT assays were performed on the endogenous GAPDH mRNA. The A0 PCR amplicon comprises the 100 bp 3′end of the RGC-32 or GAPDH 3′UTRs (no poly A tail). The position of the 100 and 200 bp DNA markers is indicated. (**B**) Diagram of the CPE and PBE4 mutant RGC-32 3′UTR psicheck 2 luciferase constructs generated by site-directed mutagenesis. (**C**) Luciferase assays using mCPE, mPBE and double mutant 3′UTR (mCPE/mPBE) luciferase constructs carried out as described in Figure 6.

Puf family proteins are known to modulate translation efficiency by interacting with deadenylases and regulating the length of the polyA tail of their target transcripts (48–50). We therefore investigated whether the loss of translational repression observed as a result of the mutation of PBE4 had any effect on polyadenylation by using extension polyA test (ePAT) assays to determine length of the polyA tail present on reporter mRNAs. Gel-based analysis of the size of the PCR product produced in ePAT assays compared to the size of the PCR product amplified from the 3′UTR cleavage site (no polyA, A0), revealed that the reporter transcripts produced in the presence of the RGC-32 3′UTR had very short polyA tails of 12–32 nucleotides in length (Figure 7A). However, when PBE4 was mutated, polyA tail length increased to a range of polyA lengths between 32 and 82 nucleotides (Figure 7A). These data therefore indicate that translational repression via PBE4 correlates with the presence of a short polyA tail and link translational repression via the RGC-32 3′UTR with deadenylation. Since CPEB can be a functional partner of Pumilio (48,51) and is involved in regulating polyA tail length, we next examined whether the putative CPE we identified through *in silico* analysis was also required for translational repression mediated by the RGC-32 3′UTR. Our results demonstrated that mutagenesis of the CPE in the wild type RGC-32 3′UTR had no effect on the ability of the 3′UTR to direct repression, indicating that the CPE is not required for 3′UTR-mediated repression (Figure 7B and C). Consistent with these observations, no further relief of repression was observed when the CPE was mutated in the context of the 3′UTR containing the PBE4 mutation (Figure 7C). These data indicate that CPEB binding to this CPE is not involved in PBE4-dependent repression.

Since co-operation between Pumilio proteins and miRNAs has been shown to play a role in regulating translational repression in different cellular contexts (52,53), we also examined the potential role of host cell miRNAs in the regulation of RGC-32 translation. We used a number of prediction programmes to identify host miRNAs that may target RGC-32. The miR-30 family (miR-30a, b, c, d, e) were the only miRNAs identified by most programmes used (Supplementary Figure S7A). Interestingly, a previous microarray analysis detected lower miR-30c and miR-30d expression in latency III cell lines compared to latency I or EBV-negative cells (54). We therefore investigated whether miR30-c and miR30-d were able to repress reporter gene expression via the RGC-32 3′UTR when they were transfected into cells. We found that these miRs reduced reporter gene expression in the presence of the RGC-32 3′UTR by 32% and 43%, respectively, when compared to a control reporter construct containing the RGC-32 ORF (Supplementary Figure S7B). In contrast, miR-30 with a mutated seed sequence did not repress reporter gene expression. However, when we mutated the miR-30 target sequence in the RGC-32 3′UTR reporter construct, we did not observe any loss of 3′UTR mediated repression in our transient transfection assays (Figure S7C). We were also unable to detect any differences in miR-30c or miR-30d expression between latency I and latency III cells using sensitive Taqman PCR assays, in contrast to the previous microarray-based study (data not shown). We therefore conclude that the miR-30 family have the potential to repress RGC-32 expression, but are not the main mediators of RGC-32 3′UTR directed repression in the B cells used in our assays.

**Figure 8.**
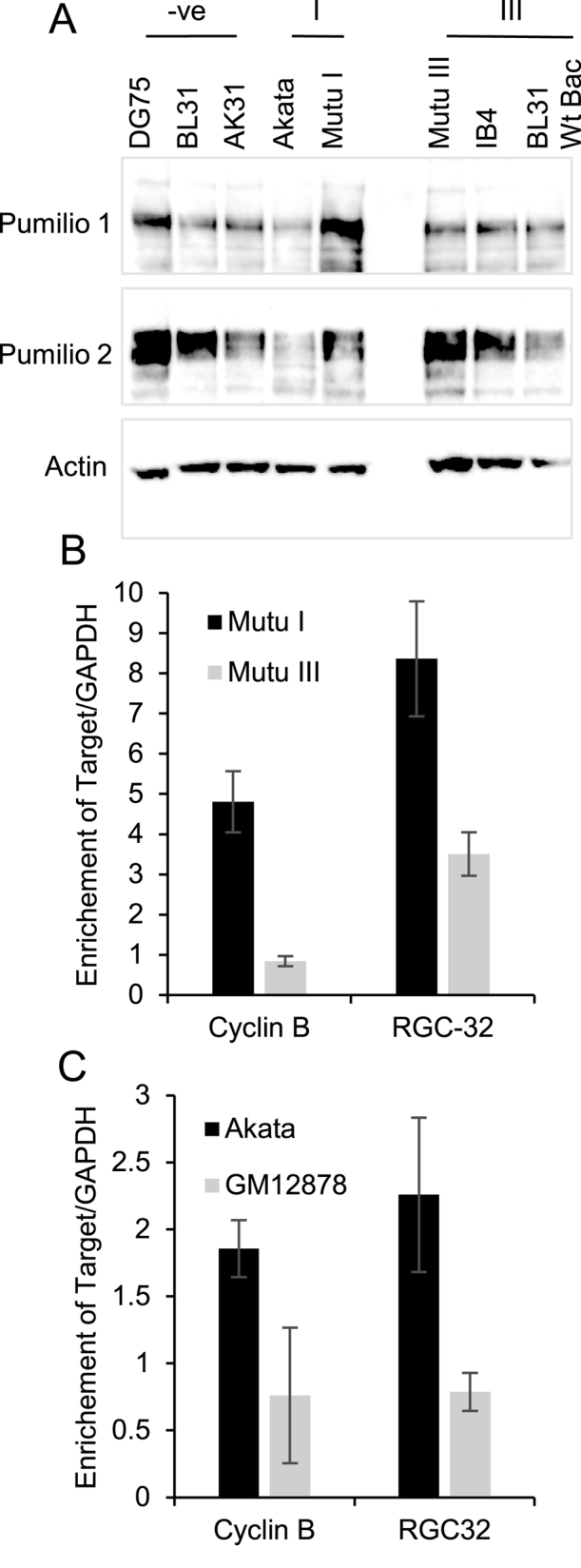
Analysis of Pumilio expression and Pumilio binding in B cell lines. (**A**) Western blot analysis of Pumilio 1 and Pumilio 2 protein expression in EBV negative (–ve), EBV-infected latency I (I) and latency III (III) cell lines. Actin levels serve as a loading control. (**B**) RNA immunoprecipitation analysis using anti-Pumilio 1 antibodies in cross-linked Mutu I (latency 1) and Mutu III (latency III) cell lines. Precipitated RNA was reverse transcribed and analysed by QPCR against a standard curve of RNA extracted from an input sample from each cell line. Percentage input values for each Pumilio 1 immunopreciptation were divided by signals obtained in the IgG control immunoprecipitation and then normalised to background enrichment for a non-target mRNA (GAPDH). Cyclin B is a known target of Pumilio and was used as a positive control for mRNA binding. Data show the mean ± standard deviation of two independent experiments. (**C**) RNA immunoprecipitation for Pumilio 1 carried out in Akata (latency I) and GM12878 (latency III) cells as in (B).

Following our identification of Pumilio proteins as regulators of RGC-32 translation, we examined whether differential expression of Pumilio proteins could explain the differential expression of RGC-32 in latency I and latency III cells. Although we found that levels of Pumilio 1 and Pumilio 2 varied across different cell lines, Pumilio proteins were not expressed at lower levels in latency III cells where RGC-32 protein expression is expressed (Figure 8A). Since Pumilio binding can be regulated by its association with other proteins and in response to different signals, we next examined whether Pumilio binding to the RGC-32 mRNA was differentially regulated in latency I and latency III cells using RNA immunoprecipitation assays. Because of the different sensitivities of latency I and latency III cell lines to lysis conditions, we performed these assays in crosslinked cells to preserve RNA-protein interactions in both cell types. We found that available Pumilio 2 antibodies did not effectively precipitate Pumilio 2 in these assays, so we were only able to analyse the level of endogenous RGC-32 mRNA precipitated using Pumilio 1 antibodies. Our data demonstrated that RGC-32 mRNA showed 2.5–3-fold lower enrichment in Pumilio 1 immunoprecipitations from latency III cells compared to latency I cells indicative of reduced Pumilio 1 binding (Figure 8B and C). This is consistent with the increased translation of RGC-32 mRNA in latency III cell lines. RGC-32 mRNA enrichment was similar or higher than the enrichment observed for the known Pumilio target mRNA cyclin B (25). Interestingly, our data demonstrated that Pumilio 1 binding to cyclin B was also reduced in latency III suggesting that the differential Pumilio 1 binding observed between these two cell types may not be RGC-32 specific. Although we detected differential binding of Pumilio 1 to cyclin B mRNA we found that cyclin B protein levels were similar in the latency I and latency III cell lines we investigated here (data not shown). Cyclin B levels are known to be transcriptionally regulated in EBV-infected cells and can also be regulated as a result of the disruption of the G2/M checkpoint by EBV-encoded latent and lytic cycle proteins so exactly how altered Pumilio binding may affect cyclin B translation is unclear (55–57). Further investigation of cyclin B regulation is however beyond the scope of the current study. Our data however indicate that reduced binding of Pumilio to the RGC-32 3′UTR may play a role in the activation of RGC-32 translation in latency III cell lines.

**Figure 9.**
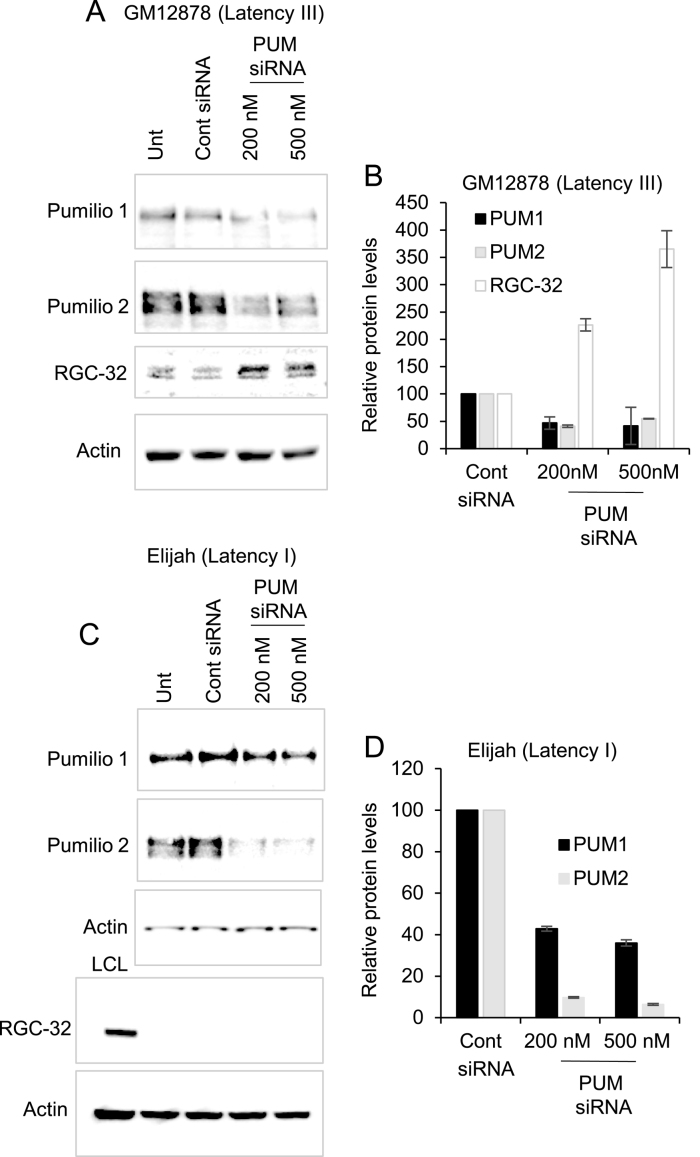
The effect of Pumilio depletion on endogenous RGC-32 expression. (**A**) Silencing of Pumilio 1 and Pumilio 2 expression in the EBV-positive GM12878 LCL (latency III) using 500 nM of non targeting control siRNA or 200 or 500 nM of a mix of Pumilio 1 and Pumilio 2 siRNAs. Cells were harvested 48 h post transfection and analysed by western blotting for Pumilio 1, Pumilio 2, RGC-32 and actin (loading control). (**B**) Quantification of the Western blot results of two independent depletion experiments in GM12878 cells. Pumilio and RGC-32 signals were normalised to the actin control and then expressed relative to the level of expression in the cells transfected with the scrambled siRNA control. Data show the mean ± standard deviation of two independent depletion experiments. (**C**) Silencing of Pumilio 1 and Pumilio 2 expression in the EBV-positive Elijah BL line (latency I) using a mix of siRNAs. Cells were transfected with siRNAs, incubated for 24 h and then re-transfected with more siRNAs and harvested after a further 24 h to achieve optimal depletion. Pumilio 1, Pumilio 2 and actin levels were determined by western blotting. Samples were re-analysed for RGC-32 expression alongside a positive control for RGC-32 expression (LCL). (**D**) Quantification of the western blot results of two independent depletion experiments in Elijah cells as in (B).

**Figure 10.**
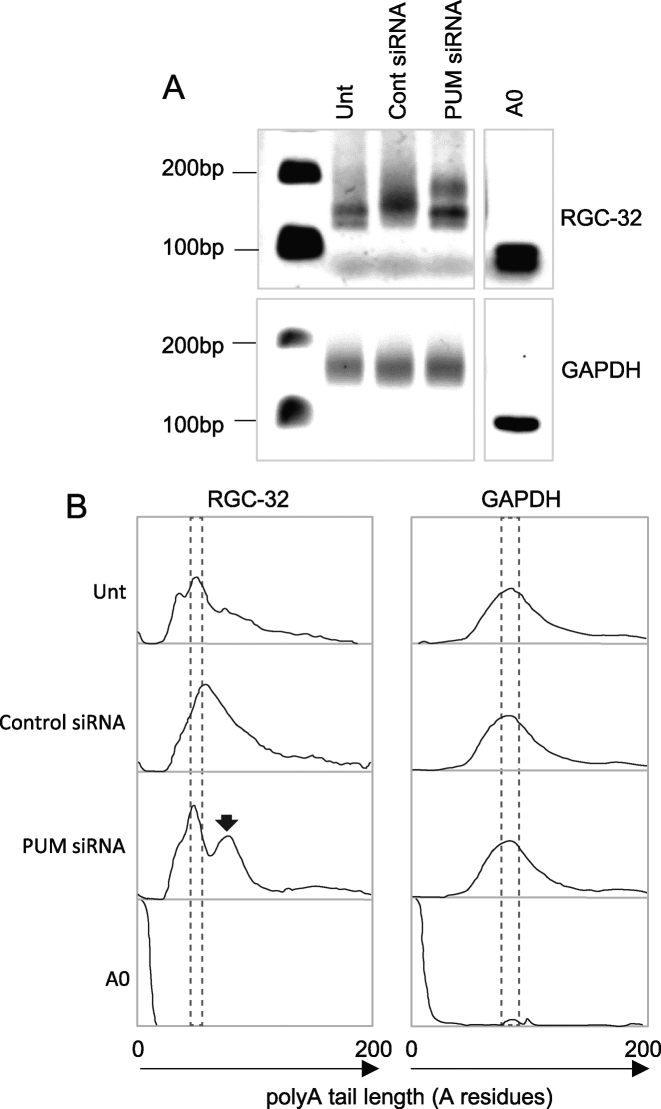
The effects of Pumilio depletion on polyadenylation of the endogenous RGC-32 mRNA. (**A**) ePAT analysis of the length of the endogenous RGC-32 mRNA polyA tail in unstransfected (Unt) GM12878 latency III cells, and in cells transfected with non targeting control siRNA (500 nM) or 200 nM of a 1:1 mix of Pumilio 1 and Pumilio 2 siRNAs. ePAT analysis of the polyA tail of the endogenous GAPDH mRNA was also measured as a control. (**B**) ImageJ quantitation of the agarose gels shown in (A). The areas boxed by dotted lines show the size of the most abundant polyadenylated mRNA species. The arrow shows the RGC-32 mRNA species with a longer polyA tail detected in cells transfected with Pumilio targeting siRNAs.

To determine whether Pumilio proteins were required for translational repression of RGC-32 *in vivo*, we knocked-down Pumilio 1 and Pumilio 2 expression in both latency I and latency III cell-lines using a pool of specific siRNAs. We found that Pumilio knock-down resulted in an up to 3.6-fold increase in RGC-32 protein expression in latency III cells (Figure 9A and B). It therefore appears that even though RGC-32 protein is expressed in latency III cells, a component of Pumilio-mediated repression is still present. This repression can be relieved by reducing Pumilio 1 and 2 expression, providing further evidence for the role of these proteins as controllers of RGC-32 translation. Interestingly, reducing Pumilio protein expression did not lead to the expression of RGC-32 protein in latency I cells (Figure 9C and D). We hypothesise that in latency I cells, Pumilio proteins initiate a translational repression event that leads to the sequestration of the RGC-32 mRNA in an inactive complex or granule that may no longer be Pumilio-dependent and may involve association with other RBPs. This hypothesis may explain why high levels of RGC-32 mRNA are detected in latency I cells. We next examined whether Pumilio knock-down in latency III cells resulted in an increase in polyA tail length. Consistent with our observations using the PBE 4 mutant 3′UTR in reporter assays, we found that knock-down of Pumilio proteins led to the appearance of endogenous RGC-32 mRNA species with longer polyA tails (Figure 10). These data therefore confirm the relationship between Pumilio-mediated RGC-32 translational repression and the presence of a short polyA tail.

## DISCUSSION

RGC-32 plays a key role in cell cycle regulation and is deregulated in numerous tumour contexts. We now show that RGC-32 is upregulated on infection of resting B cells by EBV and that a reduction in the expression of RGC-32 leads to the death of EBV-infected cells. These data therefore provide the first evidence that RGC-32 expression plays an important role in promoting the survival of EBV-immortalised cells. EBV is known to deregulate multiple cell-cycle checkpoints, including the G2/M checkpoint and we previously showed that RGC-32 overexpression in B cells leads to G2/M checkpoint disruption (15). Our data now indicate that cell-cycle regulation by RGC-32 through its role as a CDK1 activator and/or PLK1 binding protein may be crucial for the growth and survival of EBV-infected cells.

Intriguingly, RGC-32 upregulation on EBV infection of naïve B cells seems to be mediated through the relief of translational repression and occurs despite a significant reduction in RGC-32 mRNA expression, consistent with our previous observations in EBV negative and EBV-infected cell lines (15). We have now uncovered key aspects of the mechanism of this translational repression. We show that RGC-32 mRNA is associated with actively elongating ribosomes even when its translation is repressed, indicative of repression at a post-initiation stage, or perhaps simultaneous inhibition of both initiation and elongation that we would be unable to detect in our assays. We demonstrate that RGC-32 mRNA, like many cell-cycle gene mRNAs, is a target for Pumilio repressor proteins. Pumilio-mediated repression via the RGC-32 3′UTR is dependent on the presence of a single Pumilio binding site in the RGC-32 3′UTR located immediately upstream of the PAS. It is interesting that the speedy/RINGO family of atypical CDK activators are also translationally repressed through the binding of Pumilio proteins to their 3′UTRs, indicating a common mode of regulation between CDK activators across different species.

We also found that Pumilio-mediated repression correlated with the presence of a short poly A tail in reporter assays. Mutation of the essential PBE (PBE 4) in the RGC-32 3′UTR led to polyA tail lengthening of reporter mRNAs and Pumilio 1 and 2 depletion in cells led to increased RGC-32 protein expression in latency III cells and the appearance of a longer polyA tail on the endogenous RGC-32 mRNA. Our data therefore demonstrate that Pumilio-mediated repression is associated with deadenylation. Like their *C.elegans, Drosophila and* yeast PUF counterparts, human Pumilio proteins are known to repress translation in a manner that correlates with shortening of mRNA polyA tails and involves recruitment of deadenylases (49). Human Pumilio 1 and 2 proteins promote deadenylation through their association with the CCR4-NOT deadenylase complex which contains multiple subunits related to the PUF-associated yeast Pop2p and CCR4p deadenylases (21,49). In yeast, PUF-mediated deadenylation and decapping promotes mRNA degradation (58–60). The repression of RGC-32 translation we observe in EBV negative and latency I cell-lines however, does not appear to correlate with mRNA degradation, since RGC-32 mRNA levels are considerably higher in these cell-lines and in primary B cells compared to newly-infected B cells or latency III cell lines where RGC-32 is translated.

Pumilio proteins have however also been demonstrated to repress translation initiation and elongation. Repression of translation initiation may result from the effects of deadenylation on polyA tail interactions with the translation initiation machinery in the closed-loop initiation model. Pumilio-mediated inhibition of translation initiation may however also occur through competition with eIF4E for binding to the 7-methyl guanosine cap or by binding to and antagonising the function of polyA binding protein (PABP) in closed-loop translation (50,61). Our observations of the continued association of RGC-32 with polysomes in cells where its translation is repressed is however not consistent with a Pumilio-directed block to translation initiation mediated by either deadenylation or other mechanisms. Interestingly, *C.elegans* and mammalian PUF proteins have also been found to repress translation elongation through their association with the miRNA binding protein Argonaute (Ago) and the translation initiation factor, eIF1A (62). However, Ago proteins were found by another group to be dispensable for Pumilio-mediated repression by *Drosophila* and human PUFs *in vivo* (50). It is therefore possible that Pumilio-mediated repression of RGC-32 leads to the repression of translation elongation, but further work will be needed to determine whether this is Ago-dependent and involves any effects on eIF1A activity. It was recently demonstrated that deadenylation can be a secondary event in Pumilio-mediated repression (50). This study, using the RNA binding domain of Pumilio 2, found that despite the clear association between Pumilio-dependent repression and polyA tail shortening, repression was not dependent on deadenylases. Repression was however dependent on PABP. The experiments we have conducted to date cannot distinguish whether deadenylation is a cause or consequence of translational repression and perhaps the continued association of RGC-32 mRNA with polysomes points to a deadenylation-independent mechanism, but this will require further investigation.

Interestingly, we observe no differences in the total levels of Pumilio 1 and 2 in the B cell lines where RGC-32 is differentially translated, so downregulation of Pumilio expression does not appear to explain the relief of translational repression in EBV-infected latency III cell lines. We do however detect decreased binding of Pumilio 1 to both the RGC-32 and cyclin B mRNAs in latency III compared to latency I cells. This indicates that the ability of Pumilio to bind to target mRNAs is differentially controlled in EBV infected cells. How Pumilio binding may be regulated in this context is however unknown. Pumilio proteins often function in conjunction with other RNA-binding proteins like CPEB, Nanos and deleted in azoospermia-like (DAZL) in the repression of a variety of mRNAs (26,63,64) so it is possible that alterations in the levels or activity of other factors or their interactions with Pumilio are responsible for this differential binding. Identifying these putative co-operative partners will require further studies that could include screening of some known candidates, but unbiased proteomics approaches may be most informative. Pumilio 1 function can also be regulated by phosphorylation, with phosphorylation coinciding with the loss of its interaction with CPEB, so it is possible that Pumilio binding may be differentially regulated in EBV infected cells through effects on phosphorylation.

Pumilio proteins are also known to co-operate with miRNAs in the regulation of translation. For example, Pumilio 1 and 2 binding to the pre-mRNA encoding the cyclin-dependent kinase inhibitor p27 induces a conformational change required for miR-221 and miR-222-mediated repression (53). Pumilio binding to the 3′UTR of the E2F3 oncogene also enhances targeting by multiple miRNAs (52). Genome-wide studies have found that miRNA binding sites are enriched in Pumilio-targeted mRNAs with PBEs clustered around predicted miRNA binding sites (24,65). Consistent with the observations for the p27 pre-mRNA, messages with PBEs and miRNA binding sites in close proximity were found to be more likely to form stable secondary structures that can presumably be disrupted by Pumilio binding (65). We found that the miR-30 family of miRNAs were predicted to target the 3′UTR of RGC-32. Since previous microarray-based analysis had indicated that members of the miR-30 family were downregulated in latency III cells compared to latency I cells or EBV-negative cells, we examined the role of these miRs in RGC-32 translation control. Although we found that miR-30c and miR-30d could direct translational repression via the RGC-32 3′UTR in reporter assays, mutation of the miR-30 binding site in the RGC-32 3′UTR did not relieve repression mediated by the RGC-32 3′UTR. Mutation of this site did not therefore affect Pumilio-mediated repression. We were also not able to confirm previously published reports (54) of miR-30c or miR-30d downregulation in the latency III cell lines we examined. It is therefore possible that this miR family may play a role in regulating RGC-32 translation in certain circumstances, but they do not appear to co-operate in the Pumilio-mediated repression we observe here. EBV also encodes a number of viral miRNAs that could potentially play a role in RGC-32 regulation, but these miRNAs are either expressed at similar levels in latency I and latency III cells or are upregulated in latency III cells (66). This pattern of expression is the opposite expected for a directly-targeting miRNA, given that we observe activation of RGC-32 translation in latency III cells. We did not therefore investigate any potential role for these miRNAs in RGC-32 regulation.

An important question that remains outstanding concerns which EBV latent protein(s) expressed in latency III cells may mediate the changes in the host cell that leads to RGC-32 translational activation. On EBV infection, immortalised cells express the full panel of EBV latent genes that include the latent membrane proteins LMP1, 2A and 2B and the nuclear proteins EBNA-leader-protein, EBNA 1, EBNA 2, EBNA 3A, EBNA 3B and EBNA 3C. LMPs function as constitutively active receptors and activate multiple signalling pathways in infected cells and the EBNAs function as regulators of viral and cellular transcription. It will be interesting to determine whether the activity of one or more of these proteins is required to trigger the activation of RGC-32 translation through their effects on signalling or host gene expression. Infection experiments using mutant viruses and cell lines expressing individual latent proteins would be useful for future studies to elucidate this.

An interesting observation of our work concerns our inability to relieve the repression of RGC-32 translation by Pumilio protein depletion in latency I cells. This is despite our ability to increase RGC-32 translation through Pumilio depletion in latency III cells presumably through relief of some residual repression. This led us to propose that RGC-32 mRNA may be compartmentalised in a highly repressed state in latency I cells, with Pumilio depletion no longer sufficient to relieve repression. This repression likely involves additional as yet unidentified factors. Pumilio proteins are known to localise to granules containing translationally repressed mRNAs (67,68) so it is possible that Pumilio initiates a repression mechanism that involves sequestration of RGC-32 mRNA into cytoplasmic RNA granules where translation is repressed, e.g. stress granules. Consistent with our observations of repression through regulation of elongation, dendritic RNA granules have been found to contain stalled polysomes which can be reactivated rapidly (69). In preliminary immunofluorescence analysis, we have detected the presence of Pumilio proteins in granules in both latency I and latency III cells, but further studies are required to determine whether RGC-32 mRNA is present in these granules and potentially released on translational activation during infection. These experiments will be particularly challenging given the low levels of RGC-32 mRNA in the latency III cells in which the protein is translated.

In summary, we show that expression of the atypical CDK activator RGC-32 is induced on EBV infection of resting B cells and is essential for the growth of EBV-infected cells. RGC-32 protein expression is activated through the relief of translation repression, rather than through a transcriptional mechanism, a phenomenon not previously described for primary EBV infection. We reveal that the repression of RGC-32 translation is mediated by the RNA-binding protein Pumilio, is dependent on the presence of a single binding site for Pumilio in the 3′UTR and is associated with deadenylation of the RGC-32 polyA tail. Our results therefore identify a new way in which the expression of RGC-32 can be controlled that is likely to be relevant for the study of RGC-32 deregulation in numerous tumour contexts.

## Supplementary Material

Supplementary Data
