# Supplementary material for: Pumilio directs deadenylation-associated translational repression of the cyclin-dependent kinase 1 activator RGC-32
Source: Nucleic Acids Res. 2018 Jan 27;46(7):3707–25. doi: 10.1093/nar/gky038 (PMC5909466; doi:10.1093/nar/gky038)
Supplement: Supplementary Data [file gky038_supp.pdf]

	ID	Description	Sequence 5' -> 3'
PCR	MW592	SfiI RGC-32 ORF	CGGGCCTCACTGGCCTCACATACTTGCTAAAGT
	MW593	SfiI RGC-32 ORF	GCGGCCTCACTGGCCTCTAGAATGAAGCCG
Inducible shRNA	Ind shRNA-R_2	lentiviral RGC-32 shRNA 1	GATCCCCAACAGACGATCCATGCTAATATTCAAGAGATATTAGCATGGATCGTCTGTTTTTTGGAAA
	Ind shRNA-F_2	lentiviral RGC-32 shRNA 1	AGCTTTTCCAAAAAACAGACGATCCATGCTAATATCTCTTGAATATTAGCATGGATCGTCTGTTGGG
	Ind shRNA-R_4	lentiviral RGC-32 shRNA 2	GATCCCCAAAGACGTGCACTAACCTTCTTCAAGAGAGAAGGTGAGTGCACGTCTTTTTTTGGAAA
	Ind shRNA-F_4	lentiviral RGC-32 shRNA 2	AGCTTTTCCAAAAAAAAGACGTGCACTAACCTTCTCTCTTGAAGAAGGTGAGTGCACGTCTTTGGG
	MW1299	sequencing	TTGGGAATCTTATAAGTCCC
	MW1304	sequencing	AGGAATTCGAACGCTGACG
	MW1305	sequencing	TGTAAAACGACGGCCAGT
Luciferase assay	MW496	psicheck2 RGC-32 3'UTR	GCCTCGAGAACAAGAAGTTCTGGGTCCTTTC
	MW497	psicheck2 RGC-32 3'UTR-ΔDSE	CGGCGGCCGCAGACTTCAGTTCAAATTTTATT
	MW1506	psicheck2 RGC-32 3'UTR-DSE	CGGCGGCCGCGCCTCTCTCCAGCCCCC
	MW524	psicheck2 RGC-32 ORF	GCCTCGAGTCTAGAATGAAGCCGCCC
	MW525	psicheck2 RGC-32 ORF	CGGCGGCCGCTCACATACTTGCTAAAGT
	MW1430	mPBE1	ATGCTAATATACTCTTTTCTCTTAAAACATAGCTTTCCTG
	MW1431	mPBE1	GGATCGTCTGTTCTAATATG
	MW1432	mPBE2	ATAGCTTTCCACTCATTTAAAGTGCTTTTATGAAAATATTTG
	MW1433	mPBE2	GTTTTAAGAGAAAATACAATATTAGC
	MW1434	mPBE3	TGAAAATATTACTCATTAATTATATATAGTTGGAAATAGCAGTAAG
	MW1435	mPBE3	TAAAAGCACTTTAAATTACAGG
	MW1438	mPBE4	AATATATTTTACTCTACAAATAAAATTTGAACTGAAGTCTGCTC
	MW1439	mPBE4	ATAATGGGAAAGCTTACTG
	MW1442	mAU1	TTTCTGGGCACAGTCGACAGTGAAACTGACTTTGTTTAC
	MW1443	mAU1	TGGGATCAAGGGCTGATTTTAAG
	MW1444	mAU2	CTTTCCTGTACAGTCAAGTGCTTTTATGAAAATATTTG
	MW1445	mAU2	CTATGTTTTAAGAGAAAATACAATATTAG
	MW1455	mCPEB	TTTAAAGTGCTACAGAGAAAATATTTGTAATTAATTATATATAG
	MW1456	mCPEB	TTACAGGAAAGCTATGTTTTAAG
	MW1449	RGC-32 3'UTR-ΔDSE-ΔPAS	ATTTGAACTGAAGTCTGCG
	MW1450	RGC-32 3'UTR-ΔDSE-ΔPAS	TGTATACAAAAATATATTATAATGGGAAAG
	MW1446	sequencing	GGACGCTCCAGATGAAATGG
	MW588	RGC-32 3'UTR miR30 seed mt	TTAGACAGTGAAACTGACTTTGGTTCCCTGCTTGCAGCATATTAGAAC
	MW589	RGC-32 3'UTR miR30 seed mt	GTTCTAATATGCTGCAAGCAGGGAACCAAAGTCAGTTTCACTGTCTAA
3'RACE	MW86	RGC-32 forward 1	TTATAGGAACAGCTTCAGCTTC
	MW387	RGC-32 forward 2	AGCCTTCATTGCTGATCTTGA
	MW1422	Renilla luciferase forward 1	GTAACGCTGCCTCCAGCTAC
	MW1482	Renilla luciferase forward 2	GGAAATAGCAGTAAGCTTTCCCA
	3'Racemix	Degenerated primer mix	GCTCGCGAGCGCGTTTAAACGCGCACGCGTTTTTTTTTTTTTTTTTTVN
	MW1695	3'race reverse 1	GCTCGCGAGCGCGTTTAAAC
	MW1696	3'race reverse 2	GCGTTTAAACGCGCACGCGT
ePAT	MW1451	ePAT Anchor primer	GCGAGCTCCGCGGCCGCGTTTTTTTTTTTT
	MW1538	Universal reverse primer	GCGAGCTCCGCGGC
	MW1602	RGC-32 forward 2	GCTTTTATGAAAATATTTGTAATTAATTATATATAGTTGG
	MW1705	RGC-32 reverse A0	TCAGTTCAAATTTTATTTGTATAC
	MW1453	GAPDH forward 1	CCTCCAAGGAGTAAGACCC
	MW1562	GAPDH forward 2	CTCCTCACAGTTGCCATGTA
	MW1563	GAPDH reverse A0	AACTGGTTGAGCACAGGGTA
RT-QPCR	MW86*	exon 3 RGC-32	TTATAGGAACAGCTTCAGCTTC
	MW87	exon 3 RGC-32	CTGAGGAGTGACAGTGGCAG
	MW1447	β2 microglobulin forward	TTAGCTGTGCTCGCGCTACTCT
	MW1448	β2 microglobulin reverse	TGGTTCACACGGCAGGCATACT
	MW1132	CD21 forward	TCTTGGCTCTCGTCGCAC
	MW1133	CD21 reverse	TTATCACGGTACCAACAGCAATG
	MW1362	cyclin B forward	CGGGAAGTCACTGGAAACAT
	MW1363	cyclin B reverse	AAACATGGCAGTGACACCAA

Supplementary Table S1. Primer details and sequences. * previously published in Schlick *et al* 2011 but had a typographical error in the sequence so corrected here.

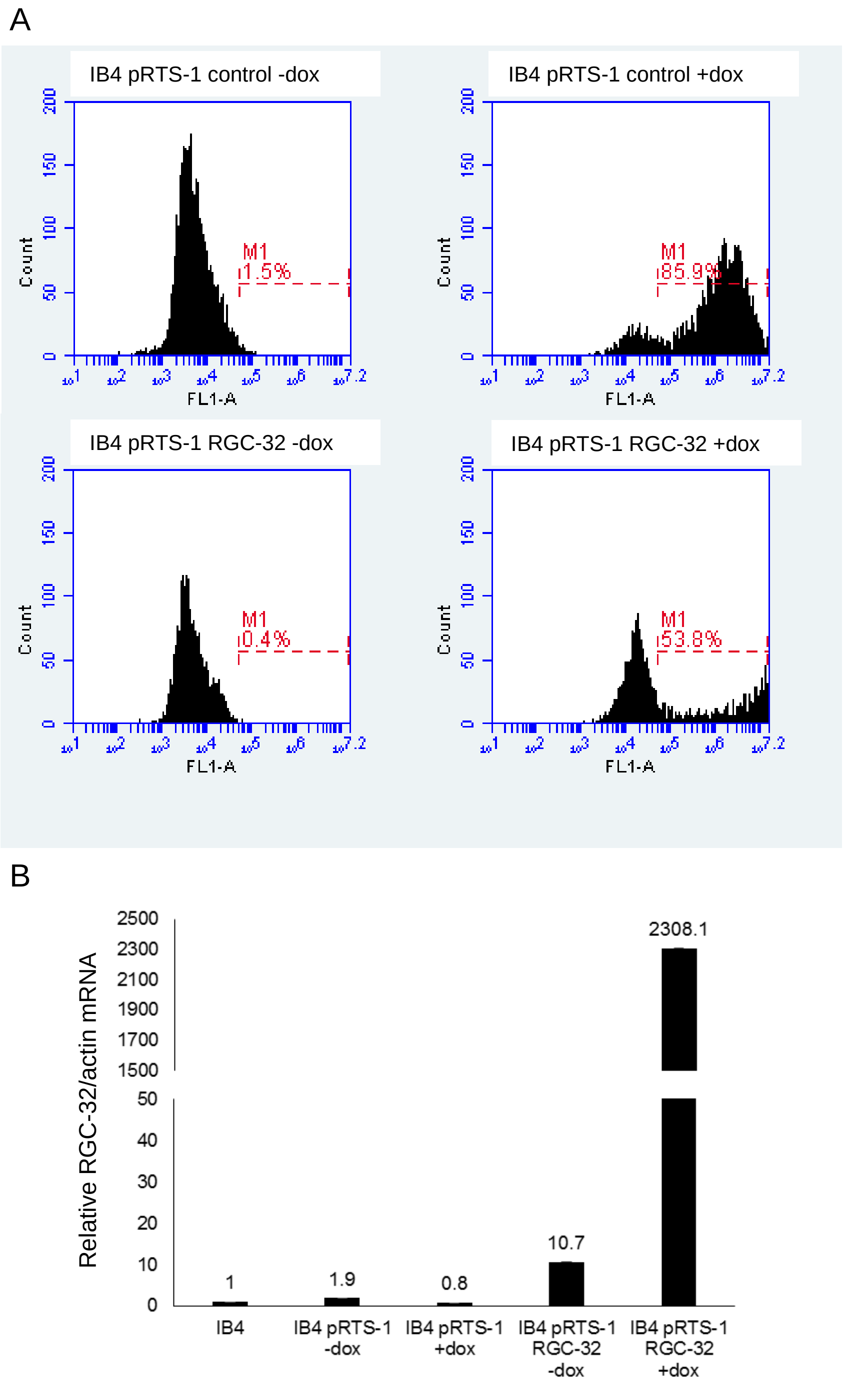

Supplementary Figure S1. Analysis of IB4 cells stably transfected with pRTS-1 plasmids that express GFP and luciferase (pRTS-1 control) or GFP and RGC-32 (pRTS-1 RGC-32) from a bidirectional doxycycline (dox) inducible promoter. (A) Flow cytometry analysis to determine the number of GFP expressing cells 48 hrs after treatment with dox. GFP positive cells were gated (M1) and the percentage of GFP positive cells is shown. GFP induction was high, but consistently less in RGC-32 expressing cells than control cells. (B) QPCR analysis of RGC-32 mRNA expression using mRNA extracted from IB4 pRTS-1 control or pRTS-1 RGC-32 cell lines 48 hrs after doxycyclin treatment. RGC-32 levels were determined by QPCR with exon 3 primers and were normalised to actin levels and expressed relative to the level in parental IB4 cells. Data show mean +/- standard deviation of PCR duplicates.

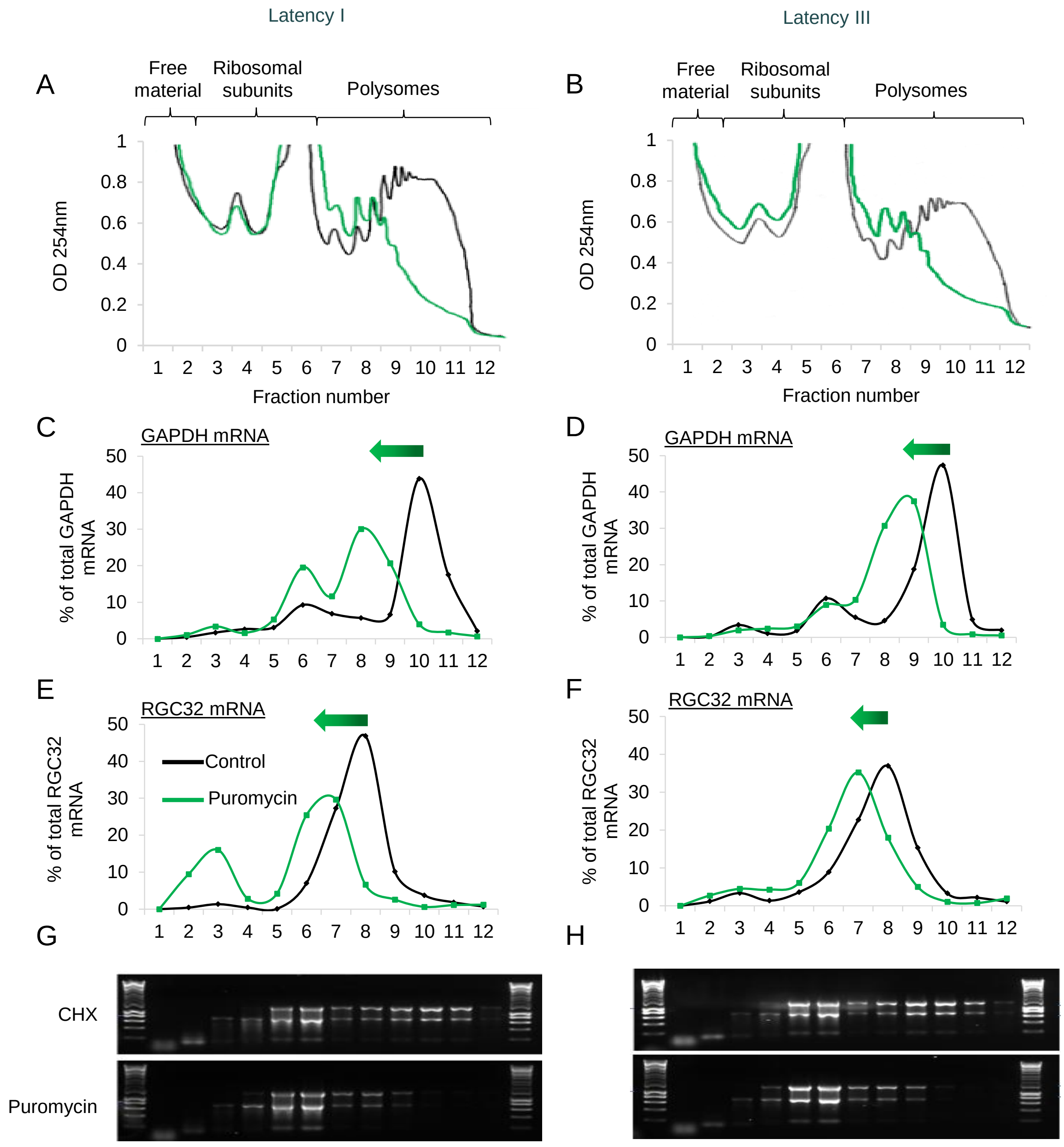

Supplementary Figure S2. Polysome gradient analysis of Mutu I cells (latency I) and Mutu III cells (latency III) treated with cycloheximide (control) or 250 µg/ml of puromycin for 15 mins prior to cycloheximide treatment. (A) and (B) show absorbance readings at 254 nm during collection of 1 ml fractions. RNA was purified from each fraction and analysed by RT QPCR for GAPDH (C and D) and RGC-32 (E and F) mRNA levels. Signals were expressed as a percentage of the total level of each mRNA across all fractions. (G and H) Agarose gel analysis of RNA samples extracted from each fraction showing the distribution of 28S and 18S ribosomal RNA species in the absence (CHX) and presence of puromycin confirming polysome disruption by puromycin treatment.

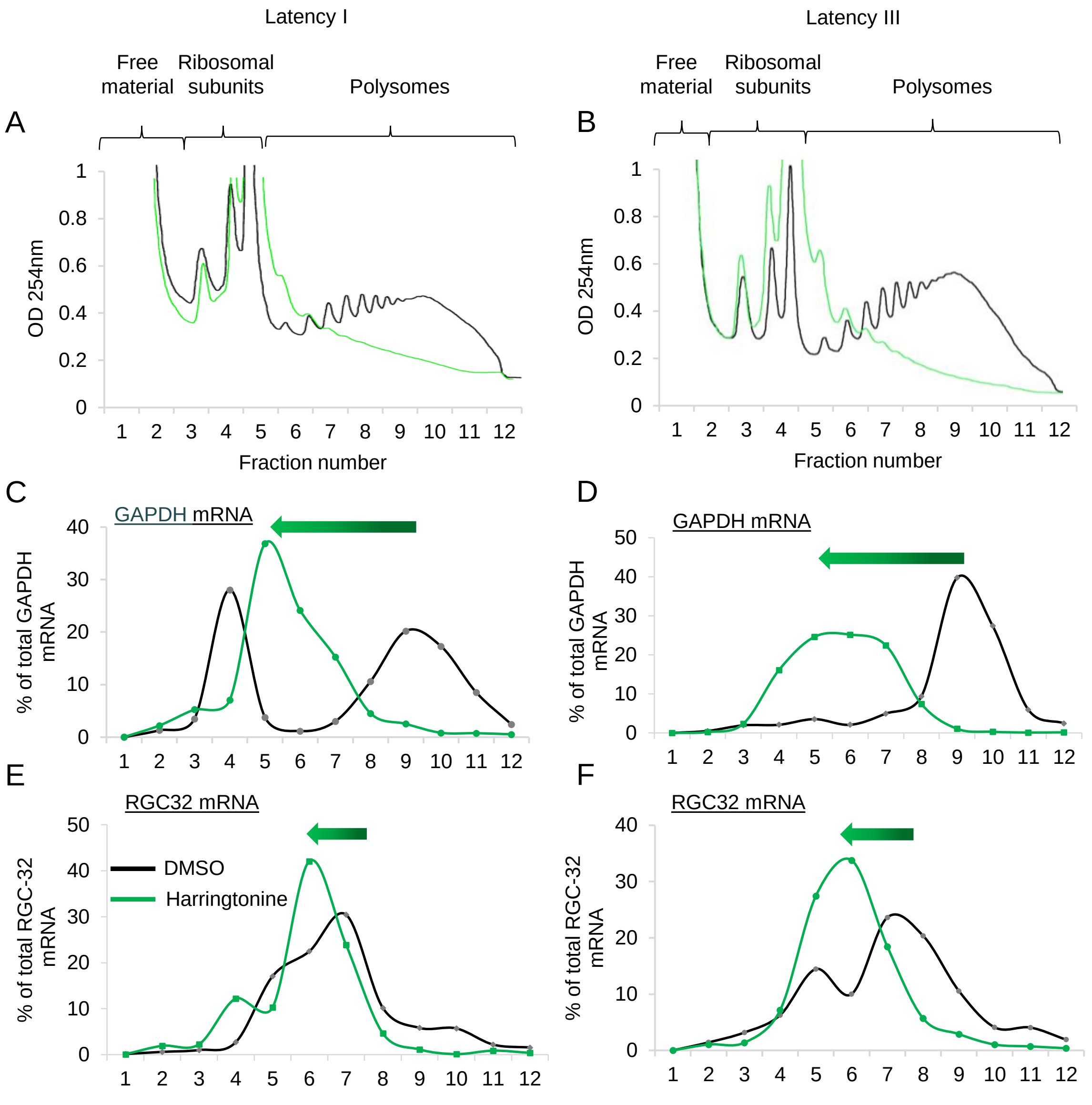

Supplementary Figure S3. Polysome gradient analysis of Akata cells (latency I) and IB4 cells (latency III) treated with 100 µg of Harringtonine or an equivalent volume of DMSO (0.1%) for 3 mins. (A) and (B) show absorbance readings at 254 nm during collection of 1 ml fractions. RNA was purified from each fraction and analysed by RT QPCR for GAPDH (C and D) and RGC-32 (E and F) mRNA levels.

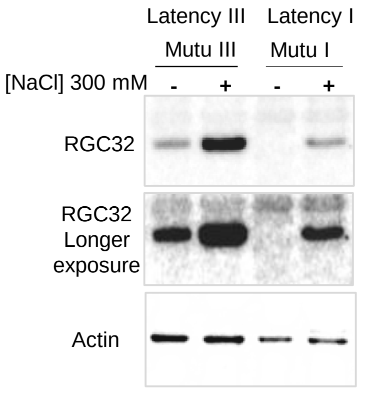

Supplementary Figure S4. Western blot analysis of RGC-32 levels in cell lysates prepared from Mutu III cells (latency III) and Mutu I cells (latency I) in the presence or absence of 300 mM Sodium Chloride. Actin levels serve as a loading control.

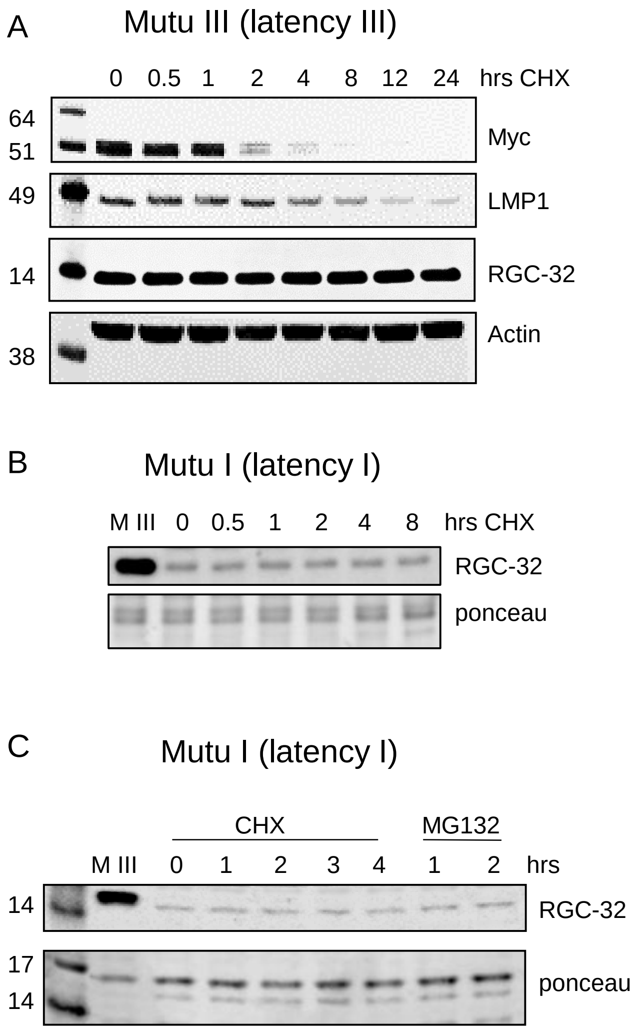

Supplementary Figure S5. Analysis of RGC-32 stability in EBV infected cell lines. (A) Mutu III cells (latency III) were incubated with 50 µg/ml cycloheximide (CHX) for the indicated time periods and cell lysates analysed by Western blotting. MYC and LMP1 are short half-life proteins and were used as controls for the effectiveness of cycloheximide treatment. Actin was used as a loading control and marker sizes (kDa) are indicated on the left. (B) Mutu I cells (latency I) were incubated with 100 µg/ml cycloheximide (CHX) for up to 8 hrs and cell lysates were analysed by Western blotting. Mutu III cell lysate (M III) was used a positive control for RGC-32 expression and ponceau S staining of the membrane was used as a loading control. (C) Mutu I cells (latency I) were incubated with 100 µg/ml cycloheximide (CHX) for up to 4 hrs or 50 µM MG132 for 1 or 2 hrs. Cell lysates were analysed by Western blotting as in (B).

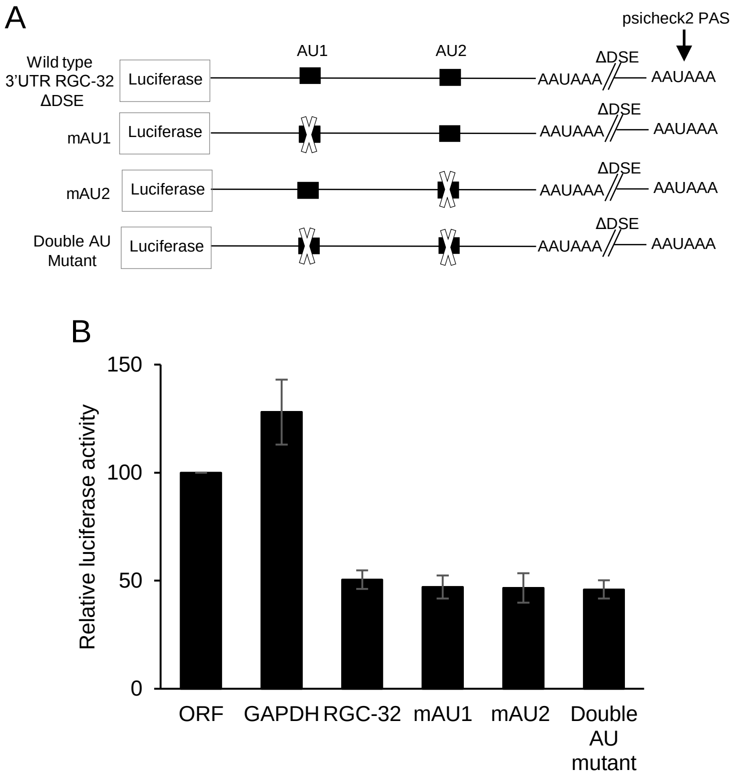

Supplementary Figure S6. Analysis of the role of putative AU-rich elements in the RGC-32 3'UTR. (A) Diagram showing the Renilla luciferase reporter constructs generated by site-directed mutagenesis. Each of the two putative AU-rich elements were mutated alone and in combination (B) Luciferase reporter assays carried out by transfecting 1 μg of each construct into DG75 cells. Results show the mean +/- standard deviation of two independent experiments normalised as in Figure 6.

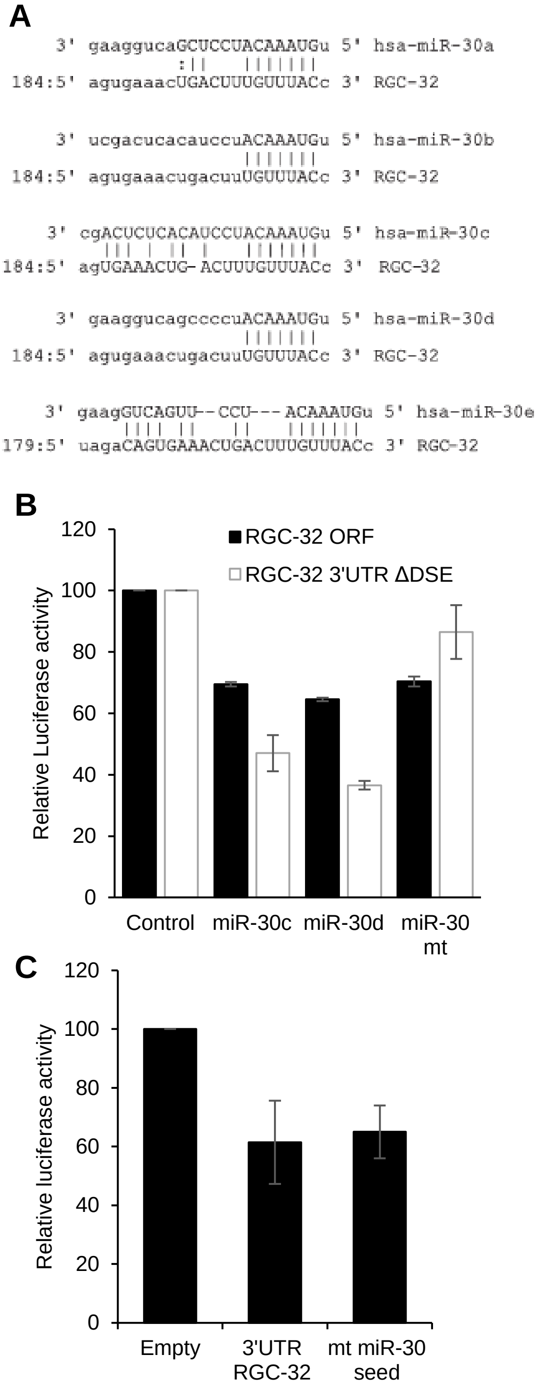

Supplementary Figure S7. Analysis of miRNA-mediated repression via the RGC-32 3'UTR. (A) miR-30a, miR-30b, miR-30c, miR-30d and miR-30e sequences and their complementarity to the RGC-32 3'UTR. (B) The effects of transfection of miR-30c, miR-30d or miR-30 mutant miRs into HeLa cells co-transfected with the psicheck2 Renilla luciferase reporter plasmid containing the RGC-32 3'UTR or the RGC-32 ORF. Renilla luciferase signals are expressed relative to the signal from control cells transfected with the psicheck luciferase reporter constructs in the absence of miRs. (C) Luciferase reporter assays using either psicheck2 empty vector, psicheck2 containing the wild-type 3'UTR or psicheck2 containing the RGC-32 3'UTR carrying mutations in the miR-30 target sequence. Results show the mean +/- standard deviation of two independent experiments normalised as in Figure 4.
